# Comparative Transcriptomic Analysis of Oral Squamous Cell Carcinoma with Progressive Cisplatin Resistance

**DOI:** 10.3390/ijms27167366

**Published:** 2026-08-18

**Authors:** Dieila Giomo de Lima, Gabriell Bonifácio Borgato, Felipe Luz Torres Silva, André Schwambach Vieira, Alessandro Santos Farias, Gustavo Narvaes Guimarães, Ana Paula de Souza

**Affiliations:** 1 Laboratory of Molecular Biology, Department of Biosciences, Piracicaba Dental School, University of Campinas—UNICAMP, Piracicaba 13414-903, SP, Brazil; dieilagl@unicamp.br (D.G.d.L.); gabriell.borgato@ceunsp.edu.br (G.B.B.); 2 Dental School, Nossa Senhora do Patrocínio Academic Center—CEUNSP, Itu 13300-200, SP, Brazil; 3 Department of Genetics, Institute of Biological Sciences, Federal University of Amazonas—UFAM, Manaus 69080-900, AM, Brazil; felipe.torres@ufam.edu.br; 4 Laboratory of Electrophysiology, Department of Neurobiology and Behavior, University of Campinas—UNICAMP, Campinas 13083-862, SP, Brazil; asv@unicamp.br; 5 Autoimmune Research Laboratory, Department of Genetics, Microbiology and Immunology, Institute of Biology, University of Campinas-UNICAMP, Campinas 13083-862, SP, Brazil; asfarias@unicamp.br (A.S.F.); gufop@unicamp.br (G.N.G.); 6 Life Sciences Core Facility (LaCTAD), University of Campinas-UNICAMP, Campinas 13083-886, SP, Brazil

**Keywords:** oral cancer, cisplatin, resistance, EMT, RNA-Seq

## Abstract

Therapy resistance remains a major cause of cancer-related mortality. Cisplatin (cis-diamminedichloroplatinum, CDDP) is a first-line chemotherapeutic agent for oral cancer, but intrinsic and acquired resistance substantially limit its clinical efficacy. Epithelial–mesenchymal transition (EMT) has been associated with drug resistance across multiple tumor types; however, its involvement in cisplatin resistance in oral cancer remains incompletely understood. Here, we established an in vitro model of cisplatin resistance using the squamous cell carcinoma 9 (SCC-9) cell line and generated two populations with distinct resistance levels. We observed morphological and transcriptional changes consistent with EMT-related features, including increased mRNA expression of *Vimentin*, *SNAIL1*, *ZEB1*, *ZEB2*, and *CDH2*. Resistant cells also exhibited reduced proliferation, whereas the intermediately resistant population, cisplatin-resistant 4 (CPR4), showed greater wound closure, suggesting enhanced migratory behavior and features consistent with a hybrid-like EMT state. Conversely, the more resistant subline, cisplatin-resistant 8 (CPR8), exhibited a more stationary phenotype. Transcriptomic profiling by RNA sequencing (RNA-seq) revealed extensive differential gene expression associated with EMT and cisplatin resistance. Functional enrichment analyses identified Gene Ontology (GO) terms related to cell adhesion, extracellular matrix organization, and calcium ion binding, while Kyoto Encyclopedia of Genes and Genomes (KEGG) pathway analysis revealed enrichment of PI3K/Akt signaling. Together, our findings support a dynamic model in which distinct EMT-related transcriptional states are associated with different levels of cisplatin resistance. This study provides insights into transcriptional changes associated with resistance acquisition and highlights candidate genes and pathways for further investigation and functional validation in oral cancer.

## 1. Introduction

Oral cavity cancer (OCC) is among the most frequent anatomical subsites of head and neck cancer (HNC) and represents a major global health burden, with nearly 390,000 new cases of lip and oral cavity cancer (2% of all cancers) and more than 188,000 deaths (1.8% of all cancers) estimated worldwide in 2022 [1]. OCC is clinically relevant due to its marked molecular and histopathological heterogeneity, aggressiveness, invasiveness, and metastatic potential [2]. Oral squamous cell carcinoma (OSCC) accounts for almost 90% of OCC cases [3].

Tobacco use, alcohol consumption, and human papillomavirus (HPV) infection are well-established risk factors for OSCC [3,4,5]. The incidence of oral cancer has increased among nonsmokers, even in the absence of HPV infection, suggesting the involvement of additional intrinsic and extrinsic factors [6,7,8].

Surgery is the standard treatment for OSCC [9]; however, metastatic disease remains a major clinical challenge [10]. Despite several advances in cancer therapy, including predictive tools supported by precision medicine, the 5-year survival rate of patients with OSCC remains approximately 50–60% [11,12].

Among the available therapeutic approaches, cisplatin (cis-diamminedichloroplatinum, CDDP)-based chemotherapy remains a first-line treatment for OSCC, either alone or combined with preoperative or postoperative radiotherapy, depending on clinical management and tumor stage [13]. CDDP is an alkylating-like agent that exerts cytotoxic effects by forming genomic DNA adducts, particularly intrastrand 1,2-cross-links between purine bases. These lesions inhibit DNA replication, disrupt cell-cycle progression, and ultimately lead to cell death [14,15]. Platinum analogues such as carboplatin and oxaliplatin have been developed to mitigate cisplatin-related toxicity, although they generally exhibit lower efficacy in this setting [16,17,18]. Cisplatin-based chemoradiotherapy therefore remains a standard treatment for locally advanced or postoperative high-risk head and neck cancer; randomized trials and large-scale meta-analyses have demonstrated improved disease control and survival compared with radiotherapy alone [19,20]. However, intrinsic or acquired resistance to platinum compounds, and in some cases multidrug resistance (MDR), substantially limits the efficacy of cisplatin-based treatment in OSCC [21,22].

Neoplastic cells exhibit substantial plasticity, enabling transitions among phenotypic states and contributing to tumor heterogeneity. Evidence suggests that this intratumoral diversity is an important contributor to therapy resistance [23,24]. Drug-tolerant cells may persist as minimal residual disease (MRD) and contribute to tumor relapse, particularly after treatment discontinuation [25].

Epithelial–mesenchymal transition (EMT) is one of the most extensively studied processes promoting tumor-cell invasion, migration, and contributing to the establishment of MRD. EMT is characterized by the loss of epithelial markers; reduced cell–cell and cell-extracellular matrix adhesion; and the acquisition of mesenchymal-like transcriptional programs, fibroblast-like morphology and cytoarchitecture, and enhanced migratory capacity [26]. This process is regulated by a network of interconnected signaling pathways, including TGF-β, WNT, and NOTCH, and by transcription factors such as SNAIL, ZEB, SLUG, and TWIST [27,28]. PI3K/Akt signaling has also been associated with EMT programming. Phosphorylated Akt can inhibit GSK-3β, leading to decreased *E-cadherin* levels in non-small cell lung cancer (NSCLC) [29]. Vimentin and N-cadherin are classical EMT markers associated with cancer stem cell (CSC) phenotypes, which may contribute to MRD through the persistence and dissemination of tumor-initiating cells [30]. Epigenetic mechanisms, including histone deacetylases and DNA methyltransferases, may further promote chromatin remodeling and DNA methylation changes that favor a CSC-like state [31].

Current evidence indicates that partial EMT (pEMT) plays a central role in tumor progression, enabling cells to acquire mesenchymal functions while retaining epithelial characteristics. Such a hybrid state may have greater metastatic potential than fully epithelial or fully mesenchymal states. Therefore, cells can occupy different transitional states with distinct molecular, morphological, and functional features [32,33,34].

Despite the extensive research on EMT and chemotherapy resistance, the relationship between distinct EMT-related states and cisplatin resistance in oral cancer remains unclear. We therefore established two populations with different levels of cisplatin resistance from a common parental OSCC cell line through stepwise drug selection. Using bulk RNA-seq, we aimed to characterize the transcriptomic changes associated with increasing cisplatin resistance and identify gene expression patterns and signaling pathways potentially involved in resistance acquisition and EMT-related cellular plasticity.

## 2. Results

### 2.1. Phenotypic Alterations During the Establishment of Cisplatin-Resistant OSCC Sublines

**Figure 1:**
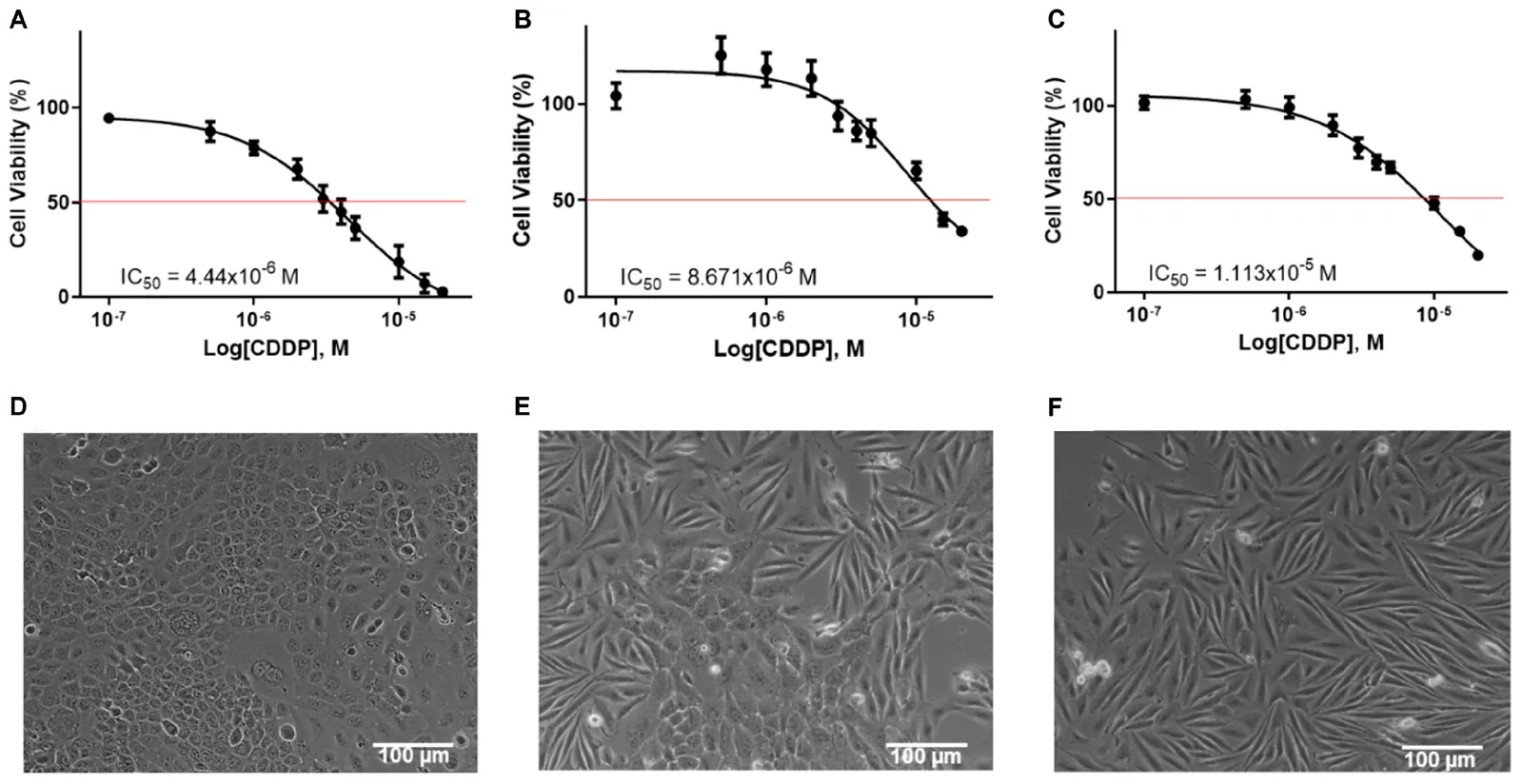
Morphological alterations and half-maximal inhibitory concentration (IC_50_) analysis following the induction of cisplatin resistance in oral squamous cell carcinoma (OSCC) cells. (**A**–**C**) IC_50_ curves showing cell viability (%) after 72 h of exposure to increasing cisplatin concentrations (M), with progressively higher IC_50_ values in the parental (P), cisplatin-resistant 4 (CPR4), and cisplatin-resistant 8 (CPR8) sublines, respectively. (**D**–**F**) Representative photomicrographs of P, CPR4, and CPR8 cells showing morphological changes associated with cisplatin resistance. Images acquired at 10× magnification; scale bar = 500 μm.

Before resistance induction, parental (P) cells had a half-maximal inhibitory concentration (IC_50_) for cisplatin of 4.44 μM (Figure 1A) and displayed a typical polygonal morphology (Figure 1D). After four intermittent rounds of treatment with 4.44 μM cisplatin, a heterogeneous cisplatin-resistant population, designated cisplatin-resistant 4 (CPR4), was generated. CPR4 was composed of both polygonal and fusiform cells and had an IC_50_ of 8.67 μM (Figure 1B,E), approximately 1.95-fold higher than that of P cells, indicating an intermediate cisplatin-resistant phenotype.

Four additional treatment–recovery cycles at the updated IC_50_ concentration of 8.67 μM generated a second resistant population, designated cisplatin-resistant 8 (CPR8). These cells displayed a fibroblast-like morphology (Figure 1F), consistent with a more pronounced mesenchymal-like phenotype, and had an IC_50_ of 11.13 μM (Figure 1C). Notably, the increase in the resistance index (RI) from 1.95-fold in CPR4 to 2.5-fold in CPR8 reflects the progressive acquisition of cisplatin resistance, accompanied by marked phenotypic changes [35].

### 2.2. Cisplatin-Resistant SCC-9 Cells Showed Reduced Growth

**Figure 2:**
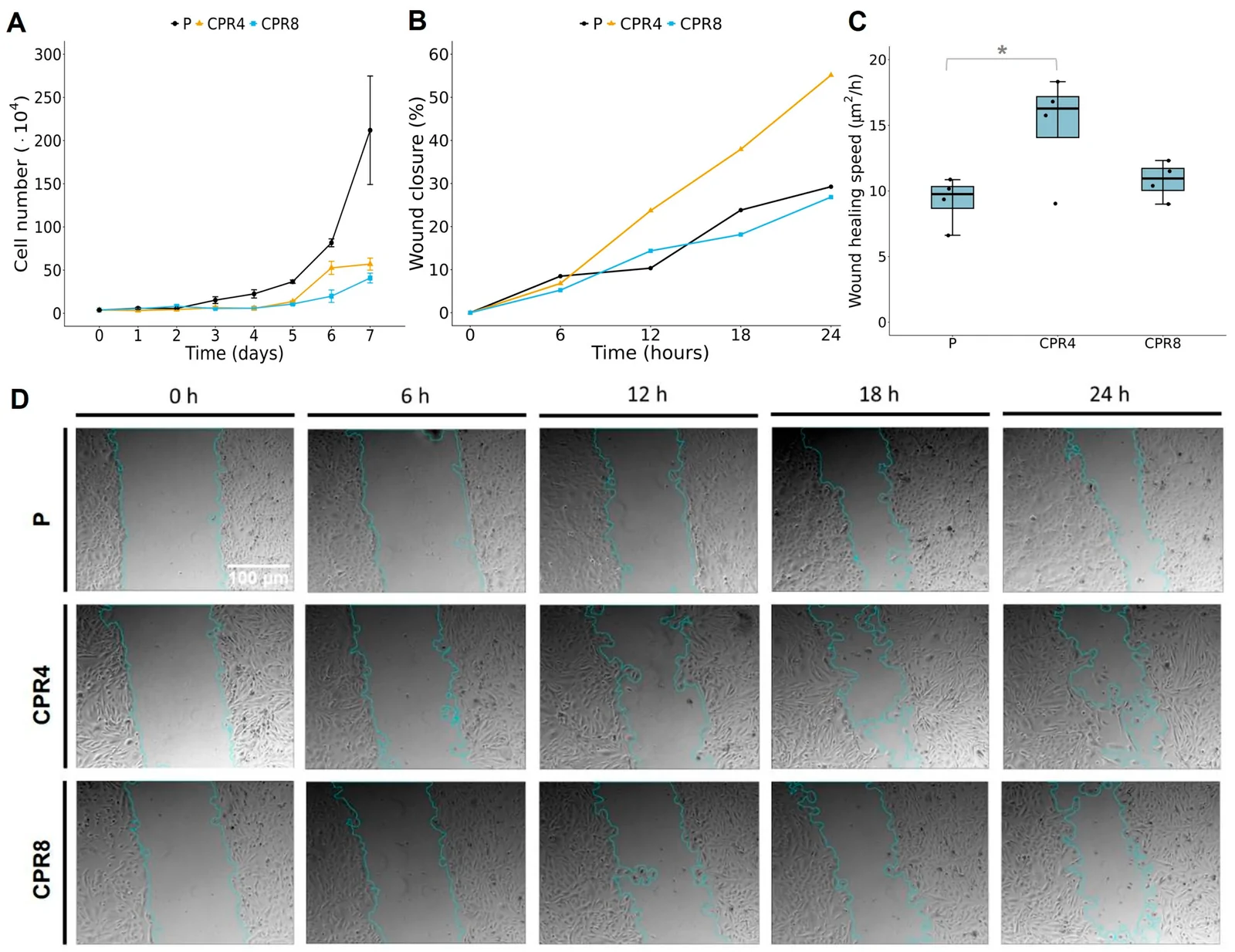
Growth curve and migratory profiles of the established cisplatin-resistant CPR4 and CPR8 (cisplatin-resistant) sublines compared with P (parental) cells. (**A**) Growth curves of P, CPR4, and CPR8 cells cultured for 7 days in cisplatin-free medium. (**B**) Percentage of the initial cell-free area covered by cells, measured at 6 h intervals over 24 h. (**C**) Wound closure rate, expressed as the reduction in scratched area over 24 h (µm^2^/h). (**D**) Representative images of the scratched areas at 0 h and wound closure by P, CPR4, and CPR8 cells at 6 h intervals over 24 h. Data are presented as the mean ± SEM; *n* = 4; * *p* < 0.05; Mann–Whitney. Scale bars represent 100 µm.

In addition to their distinct morphology, CPR cells displayed an altered growth pattern (Table 1, Figure 2A). Based on population doubling time (PDT) analysis, P cells required approximately 1.52 days to double, whereas CPR4 and CPR8 cells required approximately 2.15 and 3.41 days, respectively. These findings indicate a progressive reduction in growth rate with increasing levels of cisplatin resistance, suggesting an association between resistance acquisition and reduced proliferation.

### 2.3. CPR4 Cells Exhibited Greater Wound Closure in the Scratch Assay

To examine the association between EMT-related features and cisplatin resistance, cell migration was assessed using a scratch assay. None of the sublines completely closed the scratch within 24 h (Figure 2B). CPR4 cells showed greater wound closure than P cells, both as the percentage of wound closure (P: 30%; CPR4: 55%; *p* = 0.000379, Figure 2B) and as well as wound closure rate (P: 9 µm^2^/h; CPR4: 15 µm^2^ /h; *p* = 0.04389, Figure 2C). By contrast, CPR8 cells displayed a more stationary profile. At the wound edge, CPR4 cells exhibited elongated processes and a spindle-like morphology oriented toward the cell-free area, consistent with features of migrating cells. Taken together, CPR4 cells exhibited greater wound closure than P and CPR8 cells, suggesting enhanced migratory behavior.

### 2.4. Cisplatin-Resistant Cells Exhibited Altered Expression of EMT-Associated Markers

**Figure 3:**
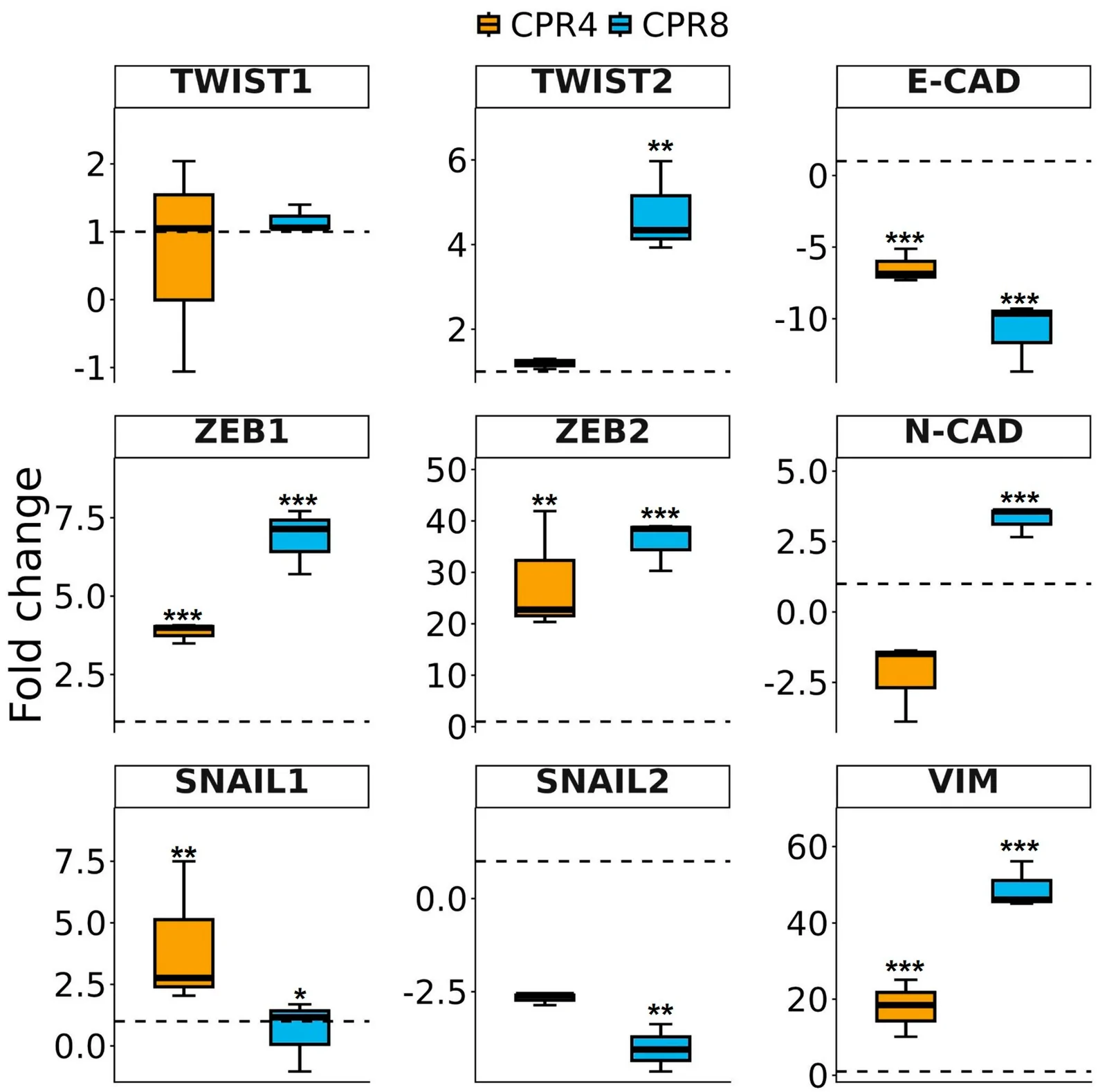
Cisplatin-resistant cells exhibit altered mRNA expression of epithelial–mesenchymal transition (EMT)-associated markers. RT-qPCR was performed to determine the relative mRNA expression levels of *TWIST1*, *TWIST2*, *E-CAD* (*CDH1*, *E-cadherin*), *ZEB1*, *ZEB2*, *N-CAD* (*CDH2*, *N-cadherin*), *SNAIL1* (*SNAIL*, *SNAI1*), *SNAIL2* (*SLUG*, *SNAI2*) and *VIM* (*Vimentin*), with *GAPDH* as the reference gene. Pairwise comparisons were performed using Student’s *t*-test. Data are presented in the box plots. * *p* < 0.05; ** *p* < 0.01; *** *p* < 0.001.

To assess whether the phenotypic alterations in cisplatin-resistant cells were accompanied by EMT-associated transcriptional changes, we evaluated the mRNA expression of canonical epithelial and mesenchymal markers in parental (P) and cisplatin-resistant (CPR4 and CPR8) cell lines (Figure 3). Both resistant sublines showed higher expression of *ZEB1*, *ZEB2*, *SNAIL1* (*SNAIL*, *SNAI1*), and *VIM* (*Vimentin*) than P cells. In CPR4 cells, expression increased by 3.85-fold for ZEB1 (*p* < 0.001), 28.33-fold for ZEB2 (*p* < 0.01), 3.33-fold for *SNAIL1* (*p* < 0.01), and 17.89-fold for VIM (*p* < 0.001). Meanwhile, in CPR8 cells, the corresponding increases for the same genes were 6.85-fold (*p* < 0.001), 35.91-fold (*p* < 0.001), 2.57-fold (*p* < 0.05), and 49.15-fold (*p* < 0.001), respectively. CPR8 also showed increased expression of the mesenchymal markers *TWIST2* and *N-CAD* (*CDH2*, *N-cadherin*). Interestingly, *SNAIL2* (*SLUG*, *SNAI2*) expression was downregulated in CPR8 cells (*p* < 0.01). This included an inverse pattern between *E-CAD* (*CDH1*, *E-cadherin*), which was downregulated, and *N-CAD* (*CDH2*, *N-cadherin*), which was upregulated, in CPR8.

### 2.5. Distinct Transcriptomic Clustering Associated with Cisplatin Resistance

**Figure 4:**
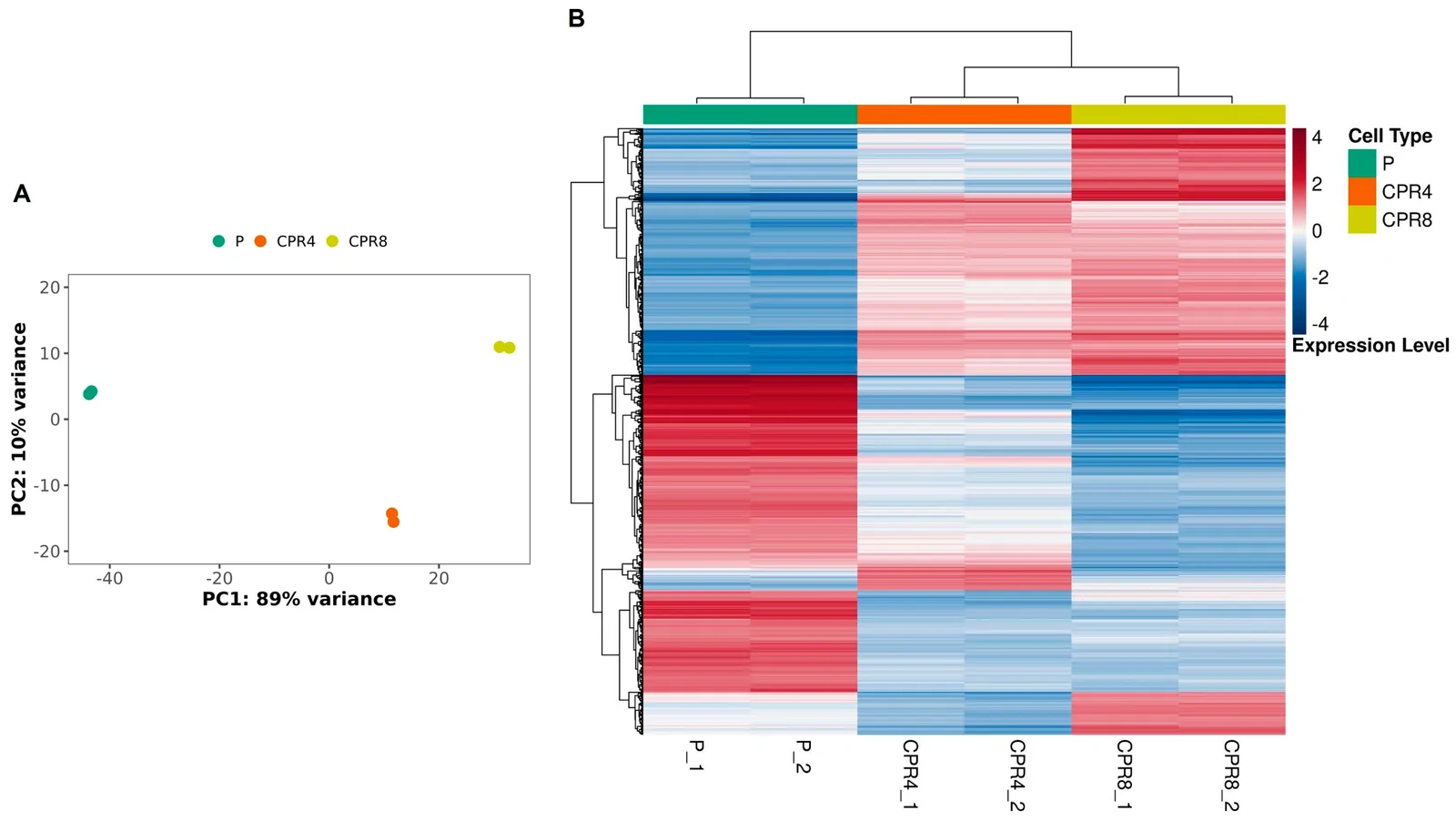
Transcriptional heterogeneity among cell sublines with different levels of cisplatin resistance. (**A**) Principal component analysis (PCA) scatter plot of RNA-seq samples from the parental (P), CPR4, and CPR8 sublines (*n* = 2 per group). Principal components 1 and 2 accounted for 89% and 10% of the total variance, respectively. (**B**) Heatmap of the 1000 most variable genes, with rows representing genes and columns representing samples, both ordered by hierarchical clustering. Columns were divided into three groups based on a three-cluster cut of the dendrogram. Group annotations are shown in green P, red CPR4, and yellow CPR8.

After quality control, prefiltering, and normalization (Figure S1A–D), principal component analysis (PCA) showed clear clustering according to cisplatin resistance levels, with replicates grouping within each condition (Figure 4A). This pattern highlights distinct transcriptional states associated with resistance acquisition.

We next visualized the expression profiles of the 1000 most variable genes in a heatmap with genes represented by rows and samples by columns; both were ordered by unsupervised hierarchical clustering (Figure 4B). The analysis identified three main clusters associated with resistance level. Importantly, CPR8 exhibited a distinct gene expression profile largely opposite to that of P, whereas CPR4 was more similar to CPR8, but retained an intermediate transcriptional profile. Small clusters with opposite patterns in CPR4 and CPR8 were also observed, indicating distinct candidate transcriptional signatures in the two resistant sublines.

### 2.6. Identification of Upregulated and Downregulated Differentially Expressed Genes

**Figure 5:**
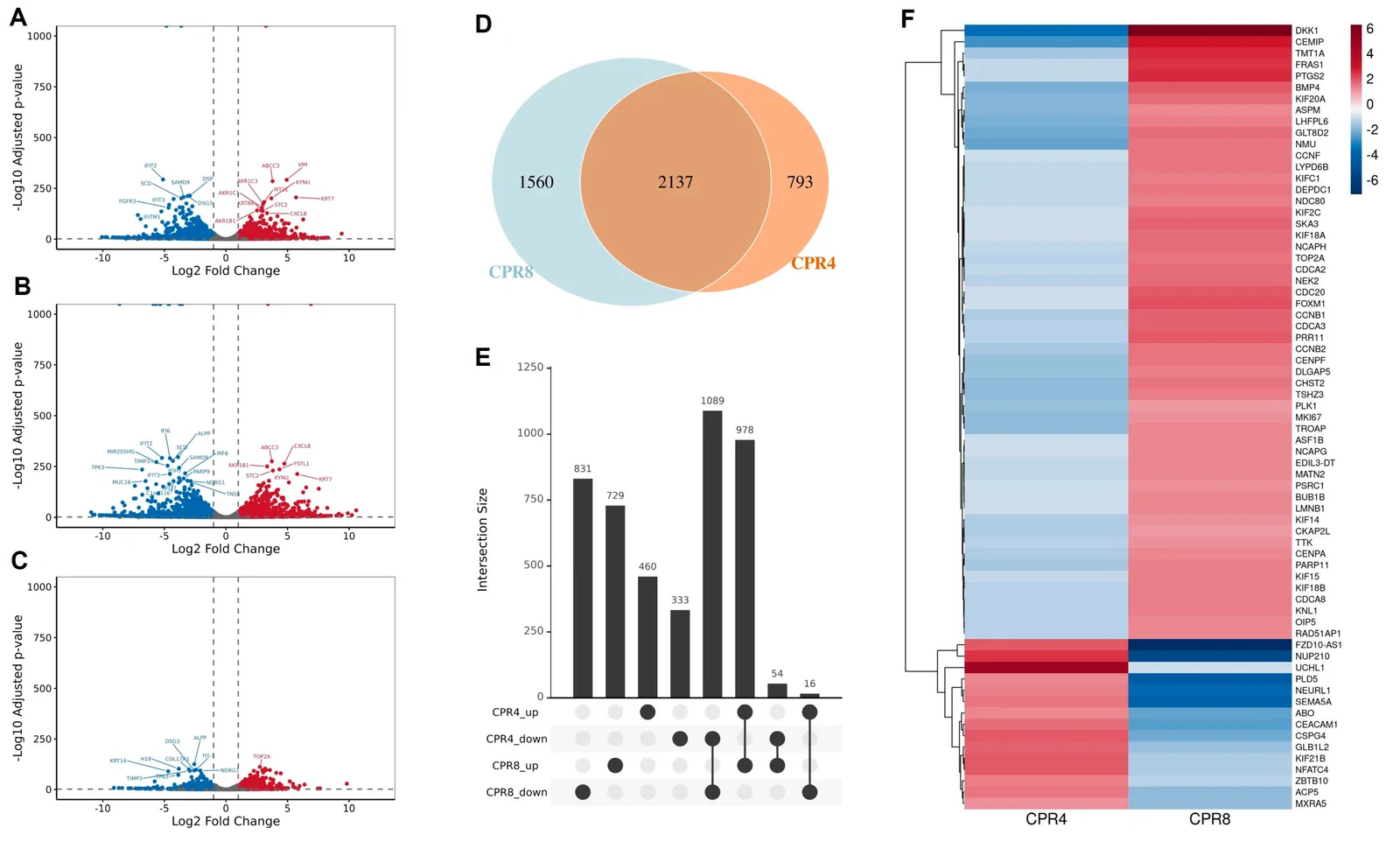
Shared and distinct differentially expressed gene (DEG) profiles in cisplatin-resistant sublines. (**A**–**C**) Volcano plot showing DEGs in each comparison. Downregulated genes are shown in green (log_2_FC ≤ −1; adjusted *p* ≤ 0.05), and upregulated genes are shown in red (log_2_FC ≥ 1; adjusted *p* ≤ 0.05), (**A**) P, (**B**) CPR4 and (**C**) CPR8. (**D**) Venn diagram showing DEGs shared by and unique to CPR4 and CPR8 relative to P. Light blue and orange indicate genes altered only in CPR8 and CPR4, respectively; dark orange indicates genes altered in both resistant sublines. (**E**) Upset plot showing combinations of upregulated and downregulated DEGs across comparisons; bar labels indicate the number of genes in each exclusive set. (**F**) Heatmap of 70 DEGs with opposite expression patterns in CPR4 and CPR8; downregulated genes are shown in blue and upregulated genes in red.

Using the predefined criteria of adjusted *p*  ≤  0.05 and log_2_FC ≤ −1 for downregulated genes or log_2_FC ≥ 1 for upregulated genes, we identified 2930 differentially expressed genes (DEGs) in CPR4 versus P (1476 downregulated and 1454 upregulated), 3697 DEGs in CPR8 versus P (1936 downregulated and 1761 upregulated), and 1672 DEGs in CPR8 versus CPR4 (848 downregulated and 824 upregulated). All comparisons showed a relatively balanced distribution of upregulated and downregulated genes (Figure 5A–C), indicating that resistance-associated transcriptional changes involved gene activation and repression to a similar extent.

To examine shared and candidate subline-specific signatures, we compared the DEGs identified in CPR4 and CPR8 relative to P. Of these, 2137 were shared by both cisplatin-resistant sublines, whereas 793 and 1560 were unique to CPR4 and CPR8, respectively (Figure 5D). Shared and unique DEGs were further stratified by direction of regulation (Figure 5E). Interestingly, fifty-four genes were downregulated in CPR4 and upregulated in CPR8, whereas 16 showed the opposite pattern (Figure 5E,F).

Among epithelial–mesenchymal transition (EMT)-associated genes, *VIM*, *ZEB1*, *ZEB2*, and *TWIST1* were upregulated in both resistant sublines, whereas *CDH1* was downregulated in both. *SNAIL2* remained unchanged relative to P (Figure S2A,B). *CDH2* and *TWIST2* were upregulated only in CPR8, whereas *SNAIL1* was upregulated only in CPR4, according to the RNA-seq differential-expression criteria (Figure S2A,B).

Direct comparison of RT-qPCR and RNA-seq results showed broad but incomplete concordance across the EMT-marker panel (Figure S2C). *ZEB1*, *ZEB2* and *VIM* increased concordantly in the resistant populations, and both methods supported increased *CDH2* and *TWIST2* in CPR8. However, *SNAI1* increased significantly in CPR4 and CPR8 by RT-qPCR but met the RNA-seq differential-expression criteria only in CPR4. *SNAI2* was significantly downregulated in CPR8 by RT-qPCR but was not significantly altered by RNA-seq. Conversely, *TWIST1* was significantly upregulated in both resistant populations by RNA-seq but was not significantly altered by RT-qPCR. Agreement between the two methods was therefore marker-dependent rather than uniform.

Taken together, these findings indicate that broad transcriptional changes accompany cisplatin resistance and that each resistant subline has a distinct candidate signature. These candidate signatures include EMT-associated genes whose expression patterns vary with resistance level, although the statistical significance of individual markers differed between analytical platforms.

### 2.7. Shared Enriched GENE Ontology-Terms in Both Resistant Cell Lines Revealed Biological Processes Associated with Cisplatin Resistance and EMT

**Figure 6:**
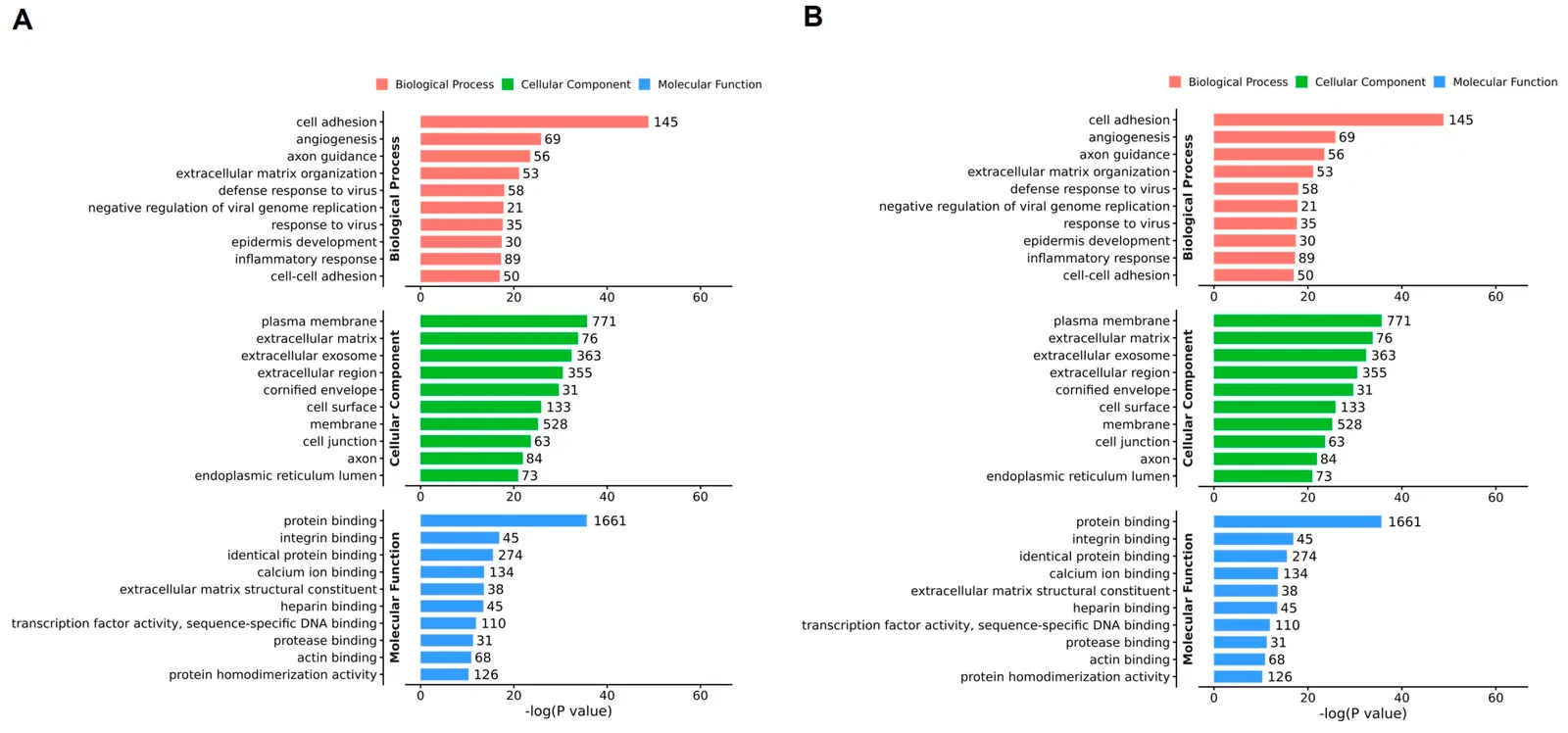
Gene Ontology (GO) terms enriched in sublines with different levels of cisplatin resistance. Bar plots show the 10 most significantly enriched GO terms for (**A**) CPR4 versus P and (**B**) CPR8 versus P. Biological process, cellular component, and molecular function terms are shown in pink, green, and blue, respectively. The number of differentially expressed genes (DEGs) associated with each term is indicated beside each bar.

To explore the potential biological roles of the identified differentially expressed genes (DEGs), we performed Gene Ontology (GO) enrichment analysis and selected the 10 most enriched terms by adjusted *p*-value. In both resistant sublines, DEGs were associated with biological processes related to cell adhesion, extracellular matrix organization, angiogenesis, and inflammatory response; cell adhesion and angiogenesis were among the most prominent terms (Figure 6A,B). These categories are closely related to epithelial–mesenchymal transition (EMT) and tumor progression [36,37,38], and are consistent with remodeling of cell–cell interactions, migration, and invasion during resistance acquisition.

In the cellular component category, terms shared by both resistant sublines included plasma membrane, extracellular matrix, and extracellular exosome (Figure 6A,B). Exosomes, a subtype of extracellular vesicles (EVs), have been implicated in cisplatin resistance, with evidence of interplay between exosome biogenesis and autophagy [39]. In some OSCC models, elevated autophagy has been associated with cisplatin resistance, and its inhibition increased drug sensitivity [39]. However, this relationship appears context-dependent, as another study found that autophagy inhibition neither restored cisplatin sensitivity nor contributed to acquired resistance in OSCC cells [40].

The most enriched molecular functions included integrin binding and calcium ion binding. Integrin binding is closely related to cell adhesion and has been widely implicated in tumor progression and resistance [41]. Calcium ion binding genes have also been associated with tumor mutation burden (TMB) in 27 of 33 TCGA cancer types [42].

Several genes contributed to multiple biological processes. The cell adhesion-related genes *CDH8*, *DSC3*, and *NECTIN1* were downregulated, whereas *ITGA4* was upregulated and associated with extracellular matrix organization in CPR4 (Figure S3A,B). MMP2 was highly expressed in both CPR4 and CPR8 and was enriched in terms related to angiogenesis, positive regulation of extracellular matrix organization, and cell migration (Figure S3A,B). This pattern suggests a potential association between *MMP2* expression and pro-invasive, EMT-related features [43].

### 2.8. PI3K/Akt Signaling Was Prominently Associated with Transcriptional Adaptation to Cisplatin Resistance

**Figure 7:**
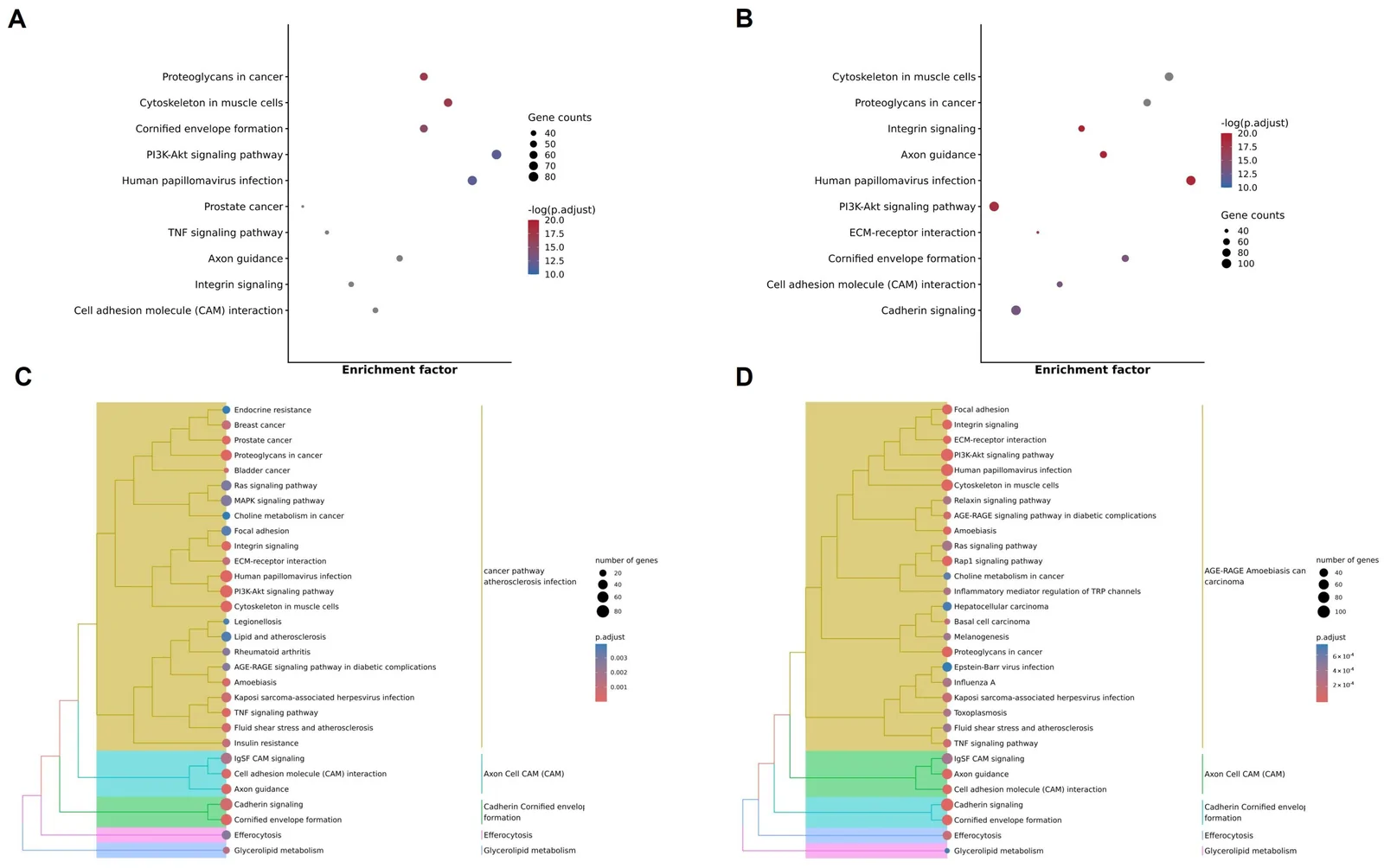
Kyoto Encyclopedia of Genes and Genomes (KEGG) pathways enriched among differentially expressed genes (DEGs). Bubble plots show the top 10 most significantly enriched pathways in (**A**) CPR4 versus P and (**B**) CPR8 versus P. Bubble size represents the number of DEGs in each pathway, color represents the adjusted p-value, the y-axis lists the pathways, and the x-axis shows the enrichment factor (number of DEGs in a pathway divided by the number of genes in the background set). Hierarchical clustering trees show enriched pathways in (**C**) CPR4 and (**D**) CPR8. Node color represents the Benjamini–Hochberg-adjusted *p*-value, and node size represents the number of DEGs in each pathway.

The differentially expressed genes (DEGs) and their corresponding log_2_FC values were subjected to Kyoto Encyclopedia of Genes and Genomes (KEGG) pathway enrichment analysis. The 10 most enriched pathways, ranked by Benjamini–Hochberg-adjusted p-values, were visualized in bubble plots (Figure 7A,B). PI3K/Akt signaling and HPV infection were among the most prominent pathways in both resistant sublines. HPV-related signaling can intersect with upstream pathways such as PI3K/Akt/mTOR [44]. However, SCC-9 is HPV negative, as confirmed by transcript-level analysis of the raw FASTQ files using HPV-KITE [45], which screens 222 HPV reference genomes. No viral sequences were detected (Figure S4). Moreover, the DEGs contributing to the HPV infection pathway were predominantly downregulated in both CPR4 and CPR8 (Figure S5A–D). Thus, enrichment of this pathway reflected coordinated repression of host genes annotated within it rather than active viral infection.

Hierarchical clustering further showed that PI3K/Akt signaling clustered closely with focal adhesion and ECM–receptor interaction pathways (Figure 7C,D). Integrin-mediated cell–ECM interactions connect these pathways by promoting focal adhesion assembly and downstream signaling, including PI3K/Akt. Such interactions regulate cytoskeletal organization and mechanotransduction and have been associated with EMT-related features and cisplatin resistance [46].

Both resistant sublines showed increased *AKT3* expression and consistent *PIK3R1* downregulation. Reduced *PIK3R1* expression, frequently observed in cancer, has been associated with decreased inhibitory control of PI3K signaling and may favor PI3K/Akt pathway activity [47]. Interestingly, HSP90 upregulation, particularly in CPR8, has also been associated with epithelial–mesenchymal transition (EMT) regulation in multiple cancer types [48]. Extracellular HSP90 (eHSP90) has been reported to promote EMT through low-density lipoprotein receptor-related protein family members, *MMP-2/9* activation, *E-cadherin* suppression, and AKT- and Wnt/β-catenin-dependent signaling [48].

Overall, these findings indicate an association between cisplatin resistance and transcriptional changes involving PI3K/Akt signaling, focal adhesion, and ECM–receptor interaction pathways, as well as altered expression of *AKT3*, *PIK3R1*, and HSP90-related genes.

### 2.9. Resistance-Associated Gene-Module Scores Increased Progressively Across SCC-9 Sublines

**Figure 8:**
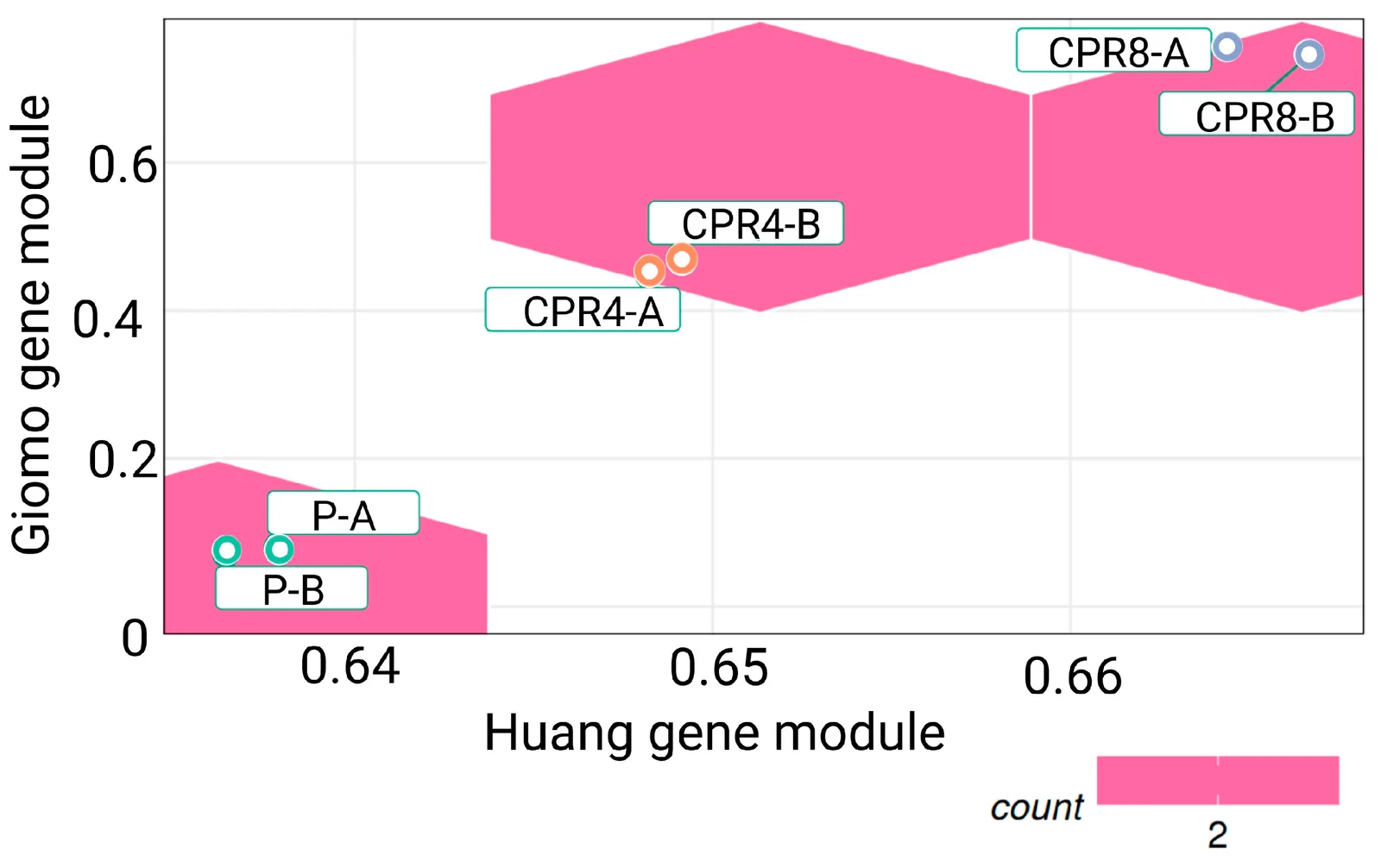
Convex hull plot of platinum-resistance module scores across parental (P), CPR4, and CPR8 cells, comparing the candidate gene set identified in this study with the gene set reported by Huang et al. [49].

Gene set scoring analysis showed that both the candidate 30-gene resistance-associated module identified in the present study and the previously reported platinum-resistance module [49] showed progressively higher scores from parental (P) to CPR4 and CPR8 (Figure 8). Within this experimental model, CPR8 had the highest scores, CPR4 an intermediate profile, and P the lowest scores. Because the candidate module was derived from and evaluated in the same limited RNA-seq dataset, this pattern should be interpreted as an exploratory description of resistance-associated transcriptional changes rather than independent validation of a predictive signature. The module comprised 30 genes, eight of which overlapped with the previously annotated platinum-resistance dataset, and may therefore reflect a combination of previously reported and model-specific features of progressive resistance.

To externally evaluate the potential clinical relevance of these modules, both gene sets were independently scored in 100 cisplatin-treated patients from the TCGA-HNSC cohort with available treatment-outcome annotations (Figure S7). Scores for the Huang platinum-resistance module differed across treatment-outcome categories, although significant pairwise differences primarily involved the treatment-ongoing group. No significant differences were detected among complete response, partial response, and progressive disease, and the module was not associated with survival status (*p* = 0.3524) (Figure S7A,C). Candidate 30-gene module scores did not differ significantly across treatment-outcome categories or survival status (*p* = 0.11) (Figure S7B,D), although deceased patients showed a nonsignificant tendency toward higher scores. Thus, the 30-gene set was retained as an exploratory candidate resistance-associated transcriptional module, providing a basis for further evaluation in independent experimental and clinical cohorts.

## 3. Discussion

In the present study, we established an in vitro model of progressive cisplatin resistance using SCC-9 cells, a tongue-derived oral squamous cell carcinoma (OSCC) cell line originating from a 25-year-old male patient. A stepwise, intermittent exposure protocol generated two resistant populations with distinct drug sensitivities, enabling comparison of morphological, proliferative, migratory, and transcriptomic changes across parental P and cisplatin-resistant derivatives (CPR4 and CPR8).

Resistance acquisition was accompanied by marked phenotypic alterations. CPR4 had an intermediate resistance level and a mixed epithelial/mesenchymal morphology, whereas the more resistant CPR8 population displayed fibroblast-like morphology and lower proliferative capacity. These observations are consistent with epithelial–mesenchymal transition (EMT) as a spectrum of intermediate or hybrid states rather than a binary process. Such states may differ in adaptive fitness, metastatic potential, and therapy resistance relative to fully epithelial or fully mesenchymal states [50].

Single-cell RNA sequencing (scRNA-seq) and assay for transposase-accessible chromatin sequencing (ATAC-seq) studies have linked EMT progression to chromatin remodeling and greater metastatic potential in hybrid states, including in mouse mammary tumors [51]. In our model, CPR4 displayed morphological and transcriptional features suggestive of an intermediate epithelial/mesenchymal state, whereas CPR8 showed features compatible with a more advanced or stabilized mesenchymal-like phenotype.

Notably, CPR4 showed greater wound closure than both the cisplatin-sensitive epithelial P cells and the more resistant CPR8 cells. Hybrid epithelial/mesenchymal (E/M) states can retain adhesive and migratory properties, integrating cell–cell adhesion and movement to favor collective migration, invasive behavior, and tumor-initiating capacity [52,53,54]. Wound closure can be influenced by proliferation. However, population doubling (PD) and population doubling time (PDT) analyses showed that both resistant sublines grew more slowly than P cells, with CPR8 showing the lowest growth rate, indicating that the greater wound closure observed in CPR4 is unlikely to be primarily explained by increased proliferation and instead provides suggestive, although not definitive, evidence of enhanced migratory behavior.

Beyond migratory behavior, EMT-marker regulation may also vary by tumor type and resistance context. *SNAIL2* has been associated with EMT and cisplatin resistance in ovarian cancer [55], but it was downregulated. However, in our model, *SNAIL2* was upregulated in CPR8 in our model, whereas *SNAIL1* was upregulated in both resistant populations. Previous data have described an inverse relationship between *SNAIL1* and *SNAIL2*, together with increased ZEB1/2 transcription during early EMT, before broader molecular and phenotypic changes become apparent [56,57].

Both resistant sublines also showed increased *ZEB1*, *ZEB2*, and *VIM* expression together with reduced *CDH1 (E-cadherin*) expression, reflecting a broader EMT-related transcriptional signature. CPR8 further showed increased *CDH2 (N-cadherin)* and *TWIST2*, indicating a more pronounced mesenchymal-like transcriptional profile than CPR4. This progression is consistent with established features of epithelial–mesenchymal plasticity, in which the cadherin switch is typically associated with more advanced stages of EMT, whereas increased *Vimentin* and EMT-associated transcription factors from the TWIST, SNAIL, and ZEB families can already be detected in earlier or hybrid states [58]. These patterns support resistance level-dependent differences in EMT-related remodeling, although they do not establish that EMT caused resistance.

EMT is primarily characterized by the loss of cell–cell adhesion and remodeling of the extracellular matrix [26,59]. Consistent with these features, GO enrichment analysis further identified biological processes related to cell adhesion, ECM organization, and angiogenesis in both resistant cell lines. Downregulation of adhesion-related genes such as Desmocollin 3 (*DSC3*), a desmosomal cadherin involved in maintaining epidermal adhesion, may reflect reduced intercellular cohesion and is consistent with reports linking low DSC3 expression to dedifferentiation and aggressive OSCC phenotypes [60]. Additionally, enrichment of integrin-binding functions and *ITGA4* upregulation likewise suggest altered cell–ECM interactions, which are critical for cell migration, survival, and proliferation. Integrin engagement can promote focal adhesion assembly and activate downstream signaling pathways, including PI3K/Akt, MAPK, and WNT, providing a potential link between ECM remodeling, intracellular survival signaling, and cisplatin resistance [41]. *MMP2* upregulation in CPR4 and CPR8 adds a transcriptional feature previously associated with ECM remodeling, invasion, metastatic dissemination, and EMT-related microenvironmental changes [43].

We also identified enrichment of calcium ion (Ca^2+^) binding functions in both cisplatin-resistant sublines, predominantly among downregulated genes (Figure 6A,B and Figure S6). Intracellular Ca^2+^ signaling contributes to cisplatin-induced apoptosis in several tumor types, including breast, ovarian, and bladder cancer [61]; thus, reduced expression of calcium ion-binding genes may be associated with diminished calcium-dependent signaling and apoptosis evasion. Although the role of calcium signaling in OSCC specifically remains poorly defined, our findings suggest that alterations in calcium ion-binding-related genes may represent an additional feature associated with cisplatin resistance. Whether this pattern contributes to resistance, relates to EMT, or represents an independent adaptation remains unknown [62].

Another pathway potentially implicated in this transcriptional remodeling is TGF-β signaling. *TGF-β1I1* and *TGF-β2* were upregulated in CPR4 and CPR8. TGF-β signaling is strongly associated with EMT-related programs, including in oral cancer [63]. This pathway is also part of a broader signaling network that interacts with noncanonical partners such as PI3K/Akt [64]. Consistent with this, our analysis revealed significant enrichment of the PI3K/Akt signaling pathway, suggesting that this pathway may be associated with transcriptional adaptation to cisplatin resistance.

The transcripts encoding the serine/threonine protein kinase AKT, a member of the AGC protein kinase family, were upregulated in both resistant cell lines, consistent with previous studies linking increased AKT signaling to chemoresistance and cancer stem cell (CSC)-related phenotypes [61]. AKT is commonly upregulated in cancer through somatic mutations or loss of *PTEN*, which normally acts as a negative regulator of the PI3K/Akt signaling [65]. Among its downstream targets, AKT can phosphorylate and inhibit TSC2, a negative regulator of mTOR, thereby favoring increased mTOR signaling; the AKT/mTOR axis is frequently altered in oral carcinogenesis, with its active form detected in approximately 50% of preneoplastic lesions, and has been associated with tumor progression and chemoresistance [66,67,68]. In addition, increased *AKT3* expression was accompanied by consistent downregulation of *PIK3R1* in both resistant cell lines. Reduced *PIK3R1* expression, frequently observed in cancer, has been associated with decreased inhibitory control over PI3K, potentially favoring PI3K/Akt pathway activation and tumorigenic processes [47].

At the pathway level, PI3K/Akt signaling was closely associated with the focal adhesion and ECM–receptor interaction pathways (Figure 7C,D). These pathways are interconnected through integrin-mediated cell–ECM interactions, which drive focal adhesion assembly and activate downstream signaling cascades. This signaling axis has also been linked to regulation of cytoskeletal organization, mechanotransduction, EMT-related features, and increased cisplatin resistance [46]. Several AKT/mTOR inhibitors, including bimiralisib, CC-115, and metformin, have undergone preclinical or clinical investigation, alone or in combination with other therapies, including cisplatin [69,70,71,72].

Interestingly, a subset, including *SEMA5A* and *FZD10-AS1*, was upregulated in CPR4 but downregulated in CPR8 (Figure 5F), indicating a resistance level-specific transcriptional pattern potentially associated with hybrid-like and more advanced EMT-related states, respectively.

*SEMA5A* has been associated with tumor invasion and metastasis through PI3K/Akt pathway activation [73], a signaling axis also implicated in the regulation of autophagy. Emerging evidence indicates a functional interplay between autophagy and exosome biogenesis in cisplatin resistance, although the role of autophagy appears to be context-dependent across OSCC models [39,40]. Because PI3K/Akt signaling and exosome-related pathways were enriched in both resistant sublines, the differential expression of *SEMA5A* suggests that its association with these pathways may vary according to the level of resistance. *SEMA5A* upregulation in CPR4 coincided with a more dynamic transcriptional profile compatible with a hybrid-like EMT-related state, whereas its downregulation in CPR8 was observed alongside a more stable, resistance-associated profile. Thus, *SEMA5A* may represent a candidate marker of resistance level-specific cellular adaptation rather than a primary regulator of these pathways.

Similarly, *FZD10-AS1* has been implicated in Wnt/β-catenin signaling through its association with *FZD10* expression, and has been linked to EMT, tumor progression, and extracellular vesicle-mediated intercellular communication associated with early and intermediate stages of tumor dissemination [74,75]. Its upregulation in CPR4 is therefore consistent with, but does not establish, a role in the hybrid-like features of this population.

Collectively, enrichment of these genes in CPR4 may reflect a transcriptional program associated with cellular plasticity, migratory capacity, and adaptability, hallmarks of hybrid-like EMT-related states, whereas their downregulation in CPR8 may accompany a more stable, stationary phenotype with a greater predominance of resistance-associated programs. This pattern is consistent with models in which hybrid E/M states show greater migratory and invasive capacity, whereas fully mesenchymal cells adopt a more stationary but therapy-resistant phenotype [32,51]. These genes therefore warrant functional investigation as candidate markers of EMT-related plasticity and cisplatin resistance.

In contrast, Dickkopf-related protein 1 (*DKK1*) was markedly upregulated in CPR8 cells and downregulated in CPR4 (Figure 5F). *DKK1* is classically described as an antagonist of canonical Wnt/β-catenin signaling and can suppress EMT in colon cancer models [76]. In cisplatin-resistant CAL27 head and neck cancer cells, *DKK1* was reduced, and its overexpression partially restored cisplatin sensitivity in that specific model [77]. However, more recent evidence associates elevated *DKK1* with cisplatin-tolerant phenotypes and macrophage-related chemoresistance in HNSCC [78], as well as with cisplatin refractoriness in non-small cell lung cancer (NSCLC) [79] and PI3K/Akt activation, EMT, and resistance in gastric cancer [80]. These context-dependent findings make *DKK1* a relevant candidate, but our data do not functionally validate its role in the SCC-9 model.

Beyond the analysis of individual genes and pathways, gene-set scoring provided an integrated view of transcriptional changes across resistance levels [81]. Despite sharing only eight genes, the candidate 30-gene module and the broader platinum-resistance module reported by Huang et al. [49] increased concordantly from P to CPR4 and CPR8 cells, suggesting that the two modules capture complementary resistance-associated transcriptional features. The limited overlap may reflect the specific cellular background and the intermittent treatment–recovery protocol used here. Because the candidate set was developed and assessed in the same small dataset, it should be regarded as exploratory rather than predictive; its smaller size, however, may facilitate future targeted evaluation using expression-based assays.

To extend this candidate module beyond the discovery dataset, external assessment in 100 cisplatin-treated patients from the TCGA-HNSC cohort did not show significant differences in candidate-module scores across treatment-response categories or survival status, although deceased patients had a nonsignificant tendency toward higher scores. This trend requires confirmation but provides a basis for investigating whether the candidate signature relates to clinically aggressive disease. HNSCC heterogeneity in anatomical site, HPV status, smoking history, and genomic background may have obscured resistance-specific associations [82]. Clinical outcomes also reflect factors not reproduced in a controlled cell-line model. The module therefore requires evaluation in independent, clinically homogeneous cohorts with well-defined cisplatin exposure and outcomes.

Despite providing important insights, this study has several limitations. First, the experimental model relied exclusively on a two-dimensional (2D) monolayer system, which does not fully recapitulate the tumor microenvironment, particularly stromal interactions, hypoxia, and immune components; whether these populations retain their cisplatin-resistant phenotype under physiologically relevant conditions remains unknown and requires validation in three-dimensional (3D) and orthotopic in vivo models. Second, only a single OSCC cell line (SCC-9) was used, and given the well-established heterogeneity of oral cancers, the generalizability of these findings to other models and clinical contexts remains limited. Third, the limited number of RNA-seq replicates restricted statistical power and the estimation of biological variability, which may partly explain the incomplete concordance between RNA-seq and RT–qPCR observed for *SNAIL1*, *SNAIL2*, and *TWIST1*; despite consistent clustering between replicates, the transcriptomic findings should therefore be considered exploratory and require evaluation in larger, independent datasets. Fourth, the study lacked functional perturbation experiments, such as pharmacological inhibition of PI3K/Akt signaling, genetic manipulation of EMT regulators, or restoration of epithelial markers followed by reassessment of cisplatin sensitivity, as well as protein-expression and phosphorylation analyses; these gaps, together with the transcriptomic and phenotypic data generated here, cannot establish causality, and the identified genes, pathways, and EMT-related states should be interpreted as hypothesis-generating rather than as definitive mechanistic evidence. Migration was assessed only by scratch assay without pharmacological inhibition of proliferation; although serum-free conditions and the slower growth of the resistant cells reduce this concern, the greater wound closure observed in CPR4 requires confirmation using mitomycin C-treated scratch assays or transwell-based methods. Finally, because RNA-seq and RT–qPCR were performed at the population level, the current data cannot determine whether epithelial and mesenchymal markers are coexpressed within individual CPR4 cells or arise from distinct subpopulations, and the external TCGA-HNSC analysis represents only an initial clinical assessment of the candidate module, warranting further evaluation in clinically homogeneous cohorts with well-defined cisplatin exposure and outcomes.

In summary, our study showed that progressive cisplatin resistance in SCC-9 cells was accompanied by distinct phenotypic and transcriptional states. Intermediate-resistant CPR4 cells showed greater wound closure and features consistent with a hybrid-like EMT-related state, whereas the more resistant CPR8 cells displayed a more pronounced mesenchymal-like phenotype, lower proliferation, and lower motility. Transcriptomic changes involved ECM organization, cell adhesion, calcium-related processes, and enrichment of PI3K/Akt signaling. These findings support associations among EMT-related plasticity, PI3K/Akt pathway-associated transcriptional alterations, and increasing cisplatin resistance; functional validation is required to establish causality. This model and dataset provide a framework for prioritizing candidate genes and pathways for mechanistic investigation.

## 4. Materials and Methods

### 4.1. Parental Cell Line Maintenance

SCC-9 cells (ATCC^®^ CRL-1629™), kindly provided by Dr. Edgar Graner (Piracicaba Dental School, University of Campinas, Piracicaba, Brazil), were cultured in DMEM/Ham’s F12 medium (1:1 mixture of Dulbecco’s modified Eagle’s medium and Ham’s F12 medium) (Gibco™, Grand Island, NY, USA) supplemented with 400 ng/mL hydrocortisone (Sigma-Aldrich, St. Louis, MO, USA), 10% fetal bovine serum (FBS) (Gibco™, Grand Island, NY, USA), antibiotic solution (100 U/mL penicillin and 100 μg/mL streptomycin) (Gibco™, Grand Island, NY, USA), and maintained at 37 °C in a humidified atmosphere containing 5% CO_2_.

### 4.2. Half-Maximal Inhibitory Concentration (IC_50_) Analysis

The anticancer drug cisplatin (Takara Bio USA, Inc., San Jose, CA, USA) was diluted in 0.9% saline solution. The cisplatin concentration used to establish resistance in the SCC-9 cells was determined by half-maximal inhibitory concentration (IC_50_) analysis. Briefly, cells were seeded in 96-well plates at a density of 4 × 10^3^ cells/well and allowed to adhere for 24 h. The cells were then exposed to cisplatin concentrations ranging from 0 to 20 µM (*n* = 8). After 72 h, cell viability was determined using the MTT assay (MTT, M6494, Invitrogen, Carlsbad, CA, USA), according to the manufacturer’s protocol. Cells cultured with vehicle alone served as the control. The IC_50_ value was calculated by regression analysis based on the percentage of viable cells at each concentration using GraphPad Prism 8 (GraphPad Software, San Diego, CA, USA).

### 4.3. Establishment of Resistant Cell Lines

Cisplatin-resistant cell populations (SCC-9CPR) were derived from the parental cell line (SCC-9P) through repeated cycles of cisplatin exposure followed by recovery periods. Initially, exponentially growing SCC-9P (P) cells were exposed to the previously determined IC_50_ concentration of cisplatin for 72 h. The medium was then replaced with fresh cisplatin-free medium, and the surviving cells were allowed to recover until their normal growth pattern was restored. This treatment–recovery cycle was repeated for a total of four rounds using the same initial IC_50_ concentration. At this stage, the first cisplatin-resistant cell population, SCC-9CPR4 (CPR4), was established. Aliquots were collected and cryopreserved for subsequent analyses, and a new IC_50_ value was determined. Thereafter, CPR4 cells were subjected to four additional cisplatin treatment–recovery cycles using the updated IC_50_ concentration, resulting in the establishment of a second resistant cell population, SCC-9CPR8 (CPR8). Aliquots were collected and cryopreserved, and a new IC_50_ value was determined. The stepwise selection process used to generate both cisplatin-resistant cell populations was conducted over approximately one year. In parallel, the parental cell line (P) was maintained under standard culture conditions throughout this period as a control. The parental SCC-9 cell line and the derived CPR4 and CPR8 populations were periodically screened for mycoplasma contamination during the resistance-selection process and were tested again after completion of cell line derivation. All cultures were confirmed to be mycoplasma-free using the MycoAlert Mycoplasma Detection Kit (LT07-318, Lonza) before the experiments reported in this study. Cell line identity was confirmed by short tandem repeat (STR) profiling.

#### 4.3.1. Measurement of Drug Resistance

Cisplatin resistance in both established cell populations, CPR4 and CPR8, was confirmed using a cell viability assay followed by IC_50_ analysis, consistent with the approach used for initial IC_50_ determination. Cells were treated with different concentrations of cisplatin for 72 h, and the resistance index (RI) was calculated as the ratio of the IC_50_ value of each cisplatin-resistant cell population to that of the parental cell line.

#### 4.3.2. Cell Morphological Analysis by Phase-Contrast Microscopy

Cell morphology was monitored using an inverted microscope (Zeiss Axiovert 40 CFL Fluorescence and Phase Contrast, ZEISS, Oberkochen, Germany), and bright-field photomicrographs were captured using ZEN 2011, Blue edition (Zeiss Software, Oberkochen, Germany).

### 4.4. Growth Curve Analysis

Cells were seeded at a density of 3.75 × 10^4^ cells/well in 24-well plates and cultured in cisplatin-free medium for 7 days. During this period, cells (*n* = 4) were detached daily by trypsinization (Sigma-Aldrich, St. Louis, MO, USA #cat. no. T4049), and viable cell numbers were determined using the Trypan Blue exclusion method. Viable cell counts were then plotted on a semi-logarithmic scale, and the population doubling level (PDL) and population doubling time (PDT) were calculated for each cell population as follows:

$$PDT=\frac{\log_{10}\left(N_{f}\right)-\log_{10}\left(N_{i}\right)}{\log_{10}\left(2\right)}$$

$$PDT=\frac{\Delta t\times \log_{10}\left(2\right)}{\log_{10}\left(N_{f}\right)-\log_{10}\left(N_{i}\right)}$$

where Ni is the number of cells seeded, Nf is the number of cells harvested at the end of the experiment, and Δt is the culture period between Ni and Nf [83].

### 4.5. Wound Healing Assay

To assess cell migration, SCC-9P and SCC-9CPR cell populations were seeded at high density in 24-well plates (*n* = 4) and cultured for 48 h until reaching 100% confluence. The confluent monolayers were scratched using a sterile 200-μL pipette tip to create cell-free areas. After washing with PBS (PBS-Gibco BRL, Life Technologies, Rockville, MD, USA) to remove detached cells and debris, the cells were cultured in fresh serum-free medium for 24 h to minimize proliferation. Wound closure was monitored by imaging of six previously selected fields every 6 h over a 24 h period using an inverted microscope (Zeiss Axiovert 40 CFL Fluorescence and Phase Contrast, ZEISS, Oberkochen, Germany). Scratch area, percentage of wound closure, and the mean and standard deviation of scratch width were quantified using the MRI Wound Healing Tool in ImageJ v1.53c [84,85]. The relative wound area was calculated by normalizing the area measured at each time point to the area measured at 0 h. In addition, the wound closure rate was calculated as the area covered over time divided by the elapsed time (h). Results are presented as mean ± SEM.

### 4.6. RNA Isolation, cDNA Synthesis and RT-qPCR

Gene expression analysis was performed by reverse transcription quantitative polymerase chain reaction (RT-qPCR). Cells were seeded at a density of 18.75 × 10^4^ cells/well in 6-well plates (*n* = 3) and cultured for 72 h. Total RNA was isolated using Trizol^®^ reagent (Invitrogen, Carlsbad, CA, USA), and its concentration and purity were assessed by spectrophotometric analysis using a NanoDrop instrument (Thermo Fisher Scientific, Waltham, MA, USA). Subsequently, 1 μg of purified total RNA was treated with DNase I (Invitrogen, Carlsbad, CA, USA) and used for cDNA synthesis with the SuperScript III First-Strand Synthesis Systems for RT-PCR (Invitrogen, Carlsbad, CA, USA), according to the manufacturer’s recommendations. Gene expression levels were determined using specific primers (Table S1) and SYBR^®^ Green PCR master mix (Applied Biosystems™, Waltham, MA, USA). Reactions were performed in a final volume of 10 μL, containing 1 μL of cDNA, 5 μL of SYBR Green PCR master mix, 150 nM of each primer, and nuclease-free water. Quantitative PCR was conducted using the 7500 Fast Real-Time PCR System (Applied Biosystems™, Waltham, MA, USA) under the following conditions: initial denaturation at 95 °C for 5 min, followed by 45 cycles of 15 s at 95 °C (denaturation), 15 s at 60 °C (annealing), and 15 s at 72 °C (extension). Target gene expression was normalized to the housekeeping reference gene glyceraldehyde-3-phosphate dehydrogenase (GAPDH) (Gene ID: 2597) (forward 5′-GTGCTGAGTATGTCGTGGAGT-3′; reverse 5′-TTGTCATATTTCTCGTGGTTCA-3′; amplicon size: 154 bp, GenBank NM_002046.3). Relative gene expression was calculated using the 2^−ΔΔCT^ method [86].

### 4.7. RNA-Seq Analysis

#### 4.7.1. Library Preparation

Following total RNA isolation from P, CPR4, and CPR8 cells (*n* = 2) using the Trizol^®^ reagent protocol (Invitrogen, Carlsbad, CA, USA), RNA concentration was determined using the Qubit™ RNA BR Assay Kit (cat. no. Q10210) on a Qubit Fluorometer (Thermo Fisher Scientific, Waltham, MA, USA), and RNA integrity was assessed using the RNA ScreenTape kit on a TapeStation 4200 system (Agilent Technologies, Santa Clara, CA, USA). RNA integrity number (RIN) values ranged from 9.7 to 9.8. A minimum input of 400 ng of total RNA was used for mRNA isolation with the NEBNext^®^ Poly(A) mRNA Magnetic Isolation Module kit (NEB, cat. no. E7490S/L, New England BioLabs, Ipswich, MA, USA), according to the manufacturer’s instructions. cDNA libraries were prepared using the VAHTS Universal V8 RNA-seq Library Prep Kit for Illumina (NR605, Vazyme, San Diego, CA, USA), according to the manufacturer’s instructions. During the ligation step, barcoded adapters from the TruSeq RNA Single Indexes—Set A kit (cat. no. 20020492, Illumina, San Diego, CA, USA) were used for sample multiplexing.

#### 4.7.2. Library Pooling and Sequencing

Final libraries were quantified using the Qubit™ dsDNA HS assay (Thermo Fisher Scientific, Waltham, MA, USA), and their size distribution and quality were assessed using the HS D1000 kit (TapeStation system, Agilent, Santa Clara, CA, USA). Libraries were pooled at equimolar concentrations, with a final loading concentration of 750 pM. Paired-end sequencing (2 × 150 bp) was performed on a NextSeq 2000 platform using the Illumina P2 Reagent kit (300 cycles) (Illumina, San Diego, CA, USA). Integrated DRAGEN BCL Convert software, version 4.2.7, was used to demultiplex the sequencing data into individual FASTQ files.

#### 4.7.3. Quality Control and Alignment

For RNA-seq analysis, the quality of the raw sequencing reads was assessed using FastQC (version 0.12.0), based on the percentage of bases with Phred quality scores above 30 (Q30). Reads were aligned to the human reference genome assembly GRCh38.p13 (hg38) using STAR version 2.7.11b (https://github.com/alexdobin/STAR (accessed on 15 January 2024)) [87] in two-pass mode.

#### 4.7.4. Differential Expression Analysis

Differential expression (DE) analysis of the bulk RNA-seq data was performed using the DESeq2 package in R version 4.6.1. Genes with an adjusted *p*-value ≤ 0.05 and a log_2_ fold change (log_2_FC) ≤ −1 were considered downregulated, whereas those with a log_2_FC ≥ 1 were considered upregulated. These thresholds were used to define differentially expressed genes (DEGs) [88]. For the matched P, CPR4, and CPR8 samples, the top 25% most highly expressed genes were selected for principal component analysis (PCA) and heatmap visualization to assess variance among P, CPR4, and CPR8 samples. The geom_mark_ellipse function (ggforce package) was used to annotate sample clusters in the PCA plot. Venn diagrams and UpSet plots were used to visualize shared and unique genes across the different comparisons.

#### 4.7.5. Functional Annotation of Differentially Expressed Genes

The Database for Annotation, Visualization and Integrated Discovery (DAVID) was used to perform functional enrichment analysis of Gene Ontology (GO) terms, including Biological Process (BP), Cellular Component (CC), and Molecular Function (MF), based on lists of differentially expressed genes (CPR4 or CPR8 vs. P) ranked by fold-change. To further explore the functional implications of these differentially expressed genes (DEGs), pathway enrichment analysis was conducted using the Kyoto Encyclopedia of Genes and Genomes (KEGG) database. KEGG pathway enrichment analysis was performed in the R environment using the clusterProfiler ecosystem for enrichment analysis and visualization [89].

#### 4.7.6. Gene Set Scoring and External Clinical Evaluation

Genes associated with the resistant phenotype were selected using stringent differential expression criteria (log_2_FC > 5 and FDR < 0.01). Gene set scoring was performed using the singscore package, version 1.22.0 [81]. Two predefined gene modules were scored independently: the candidate 30-gene resistance-associated module identified in the present study and the annotated set of 812 genes previously associated with platinum resistance, as reported by Huang et al. [49].

To externally evaluate the potential clinical relevance of these modules, both gene sets were applied to a cohort of patients with head and neck squamous cell carcinoma from the TCGA-HNSC project. Only patients who had received cisplatin-based therapy and had available treatment-outcome annotations were included; a total of 100 patients met these criteria. Module scores were calculated for each patient using singscore and compared across treatment-response categories and survival status.

### 4.8. Statistical Analysis

Statistical analysis of the growth curve, wound closure assay, and RT-qPCR gene expression was conducted in the R environment (R version 4.3.1). Comparisons between two groups in RT-qPCR data were performed using a two-tailed Student’s *t*-test. All tests were conducted using a significance level of 0.05, and the results are reported with their corresponding *p*-values.

## Figures and Tables

**Table 1:** Growth characteristics of parental (P) and cisplatin-resistant (CPR4 and CPR8) cell sublines.

Cell Line	Population Doubling	Population Doubling Time (Days)
P	5.25	1.52
CPR4	3.73	2.15
CPR8	2.35	3.41

## Data Availability

The data that support the findings of this study are available from the corresponding author upon reasonable request.

## Supplementary Materials

The following supporting information can be downloaded at https://www.mdpi.com/article/10.3390/ijms27167366/s1.
