# Supplementary material for: Comparative Transcriptomic Analysis of Oral Squamous Cell Carcinoma with Progressive Cisplatin Resistance
Source: Int J Mol Sci. 2026 Aug 18;27(16):7366. doi: 10.3390/ijms27167366 (PMC13513681; doi:10.3390/ijms27167366)
Supplement: Supplementary file 1 [file ijms-27-07366-s001.zip › ijms-4452391-supplementary.pdf]

Supplementary Figures

Figure S1

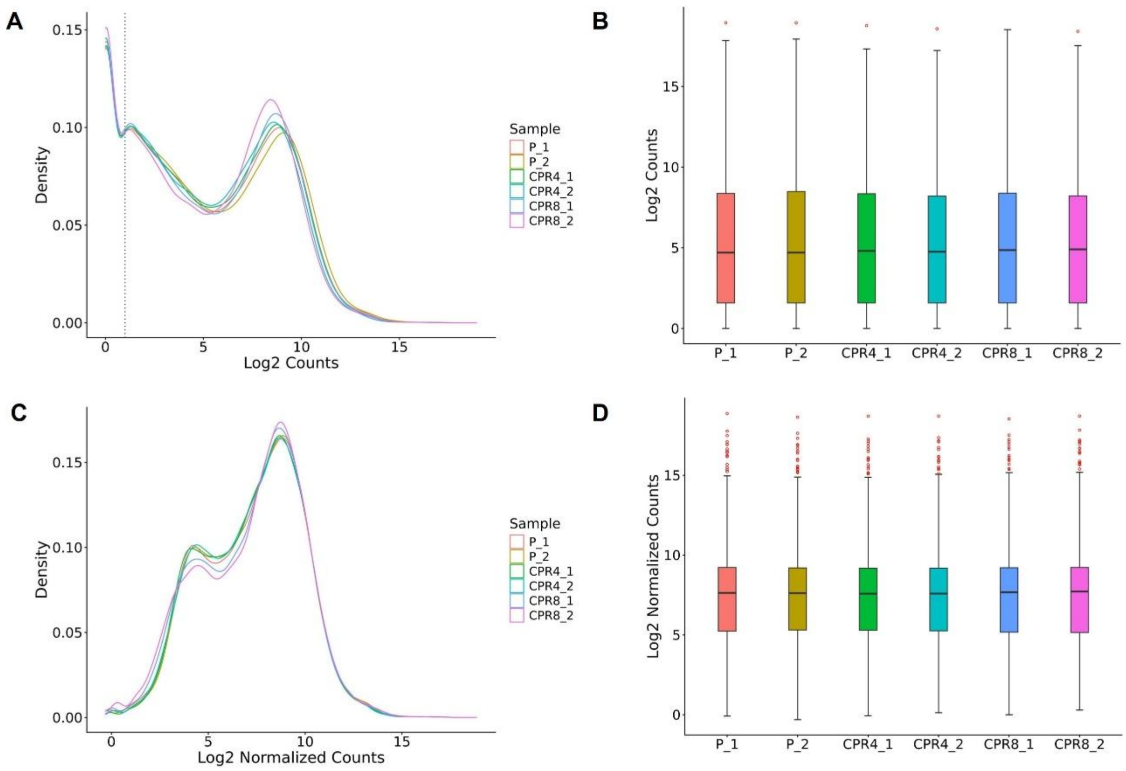

Figure S1. Initial parameters from DESeq2. (**A**) Density graph and boxplot of raw counts (log2) per sample. (**B**) Density graph and boxplot of counts (log2) filtered and normalized per sample.

Figure S2

A B

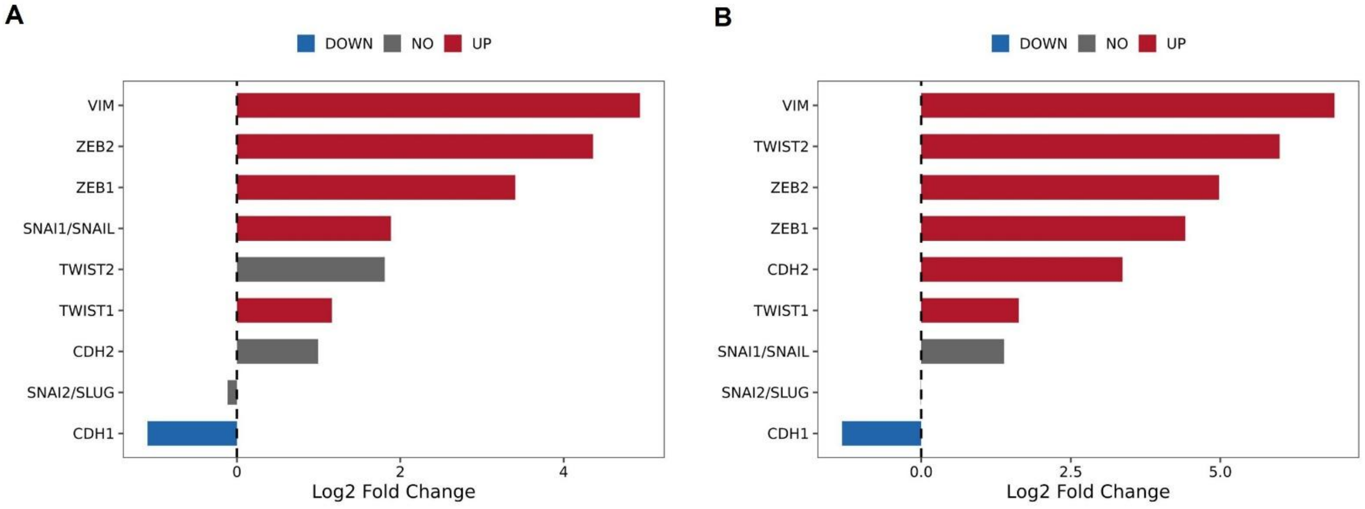

C

Gene	CPR4 RT-qPCR	CPR4 RNA-seq	CPR8 RT-qPCR	CPR8 RNA-seq	Assay Concordance
ZEB1	↑ Increased	↑ Upregulated	↑ Increased	↑ Upregulated	✓
ZEB2	↑ Increased	↑ Upregulated	↑ Increased	↑ Upregulated	✓
VIM	↑ Increased	↑ Upregulated	↑ Increased	↑ Upregulated	✓
E-CAD/CDH1	↓ Decreased	↓ Downregulated	↓ Decreased	↓ Downregulated	✓
N-CAD/CDH2	No change	No change	↑ Increased	↑ Upregulated	✓
TWIST1	No change	↑ Upregulated	No change	↑ Upregulated	●
TWIST2	No change	No change	↑ Increased	↑ Upregulated	✓
SNAIL1/SNAI1/SNAIL	↑ Increased	↑ Upregulated	↑ Increased	No change	✓ CPR4 | CPR8 ●
SNAIL2/SNAI2/SLUG	No change	No change	↓ Decreased	No change	✓ CPR4 | CPR8 ●

↑ =Increased / Upregulated ↓ = Decreased / Downregulated ✓ = Concordant ● = Assay-dependent

Figure S2. Identification of differentially expressed genes (DEGs) profiling 2xpressio-mesenchymal transition (EMT). Bar graphs showing the most frequent EMT DEGs *Vimentin* (*VIM*), *ZEB1*, *ZEB2*, *SNAI1* (*SNAIL1*/*SNAIL*), *SNAI2* (*SLUG*/*SNAIL2*), *TWIST1*, *TWIST2*, *CDH1* (*E-CAD*/*E-cadherin*) and *CDH2* (*N-CAD*/*N-cadherin*) downregulated in blue, upregulated in red and without changes in grey in both groups (**A**) CPR4 and (**B**) CPR8. Concordance between RT-qPCR and RNA-seq 2xpression. patterns for EMT-related markers in cisplatin-resistant SCC-9 sublines. (**C**) Expression changes detected by RT-qPCR and RNA-seq are summarized for CPR4 and CPR8 cells relative to parental SCC-9 cells. Assay-dependent results indicate discordant significance or 2xpression. patterns between the two methods. Genes showing concordance in only one resistant subline are indicated accordingly.

Figure S3

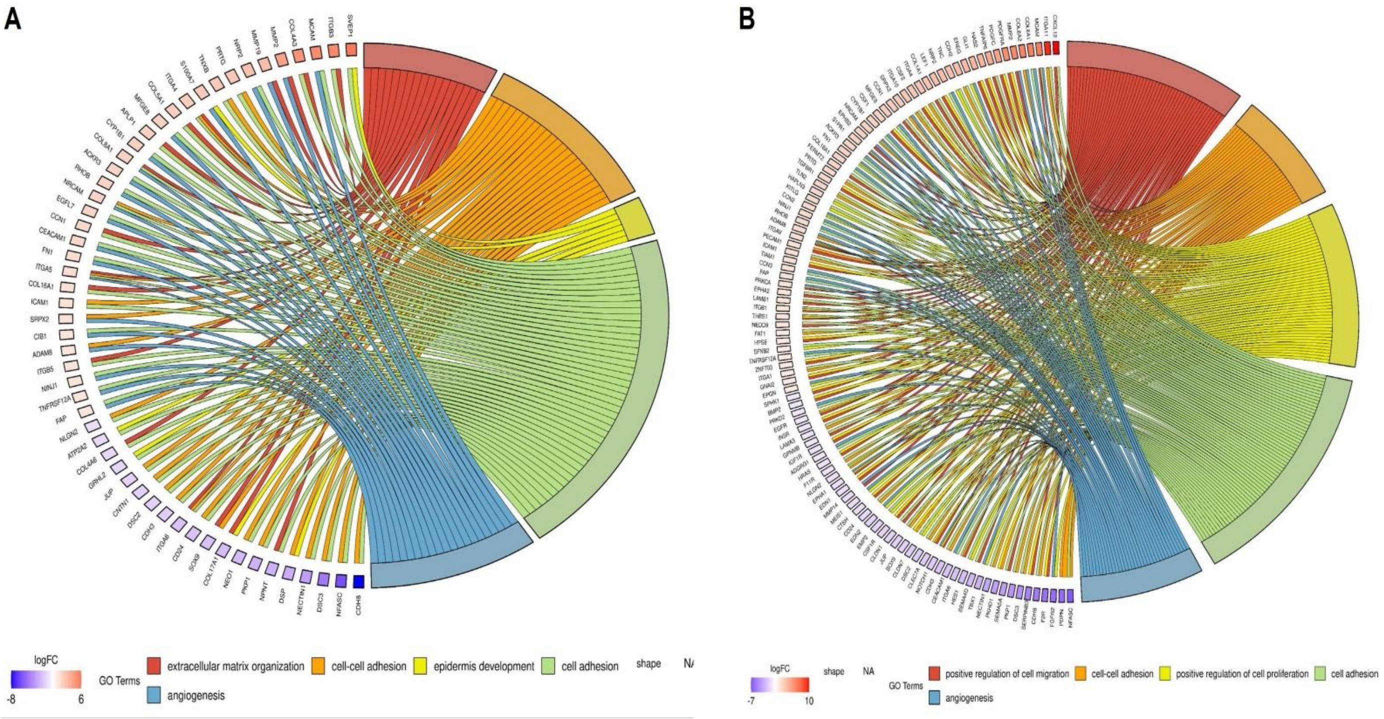

Figure S3. Chord plot with genes involved/shared in five biological process Gene Ontology-terms (GO-terms) enriched, being to (**A**) CPR4: extracellular matrix organization, cell-cell adhesion, epidermis development, cell adhesion, angiogenesis and (**B**) CPR8: positive regulation of cell migration, cell-cell adhesion, positive regulation of cell proliferation, cell adhesion and angiogenesis.

Figure S4

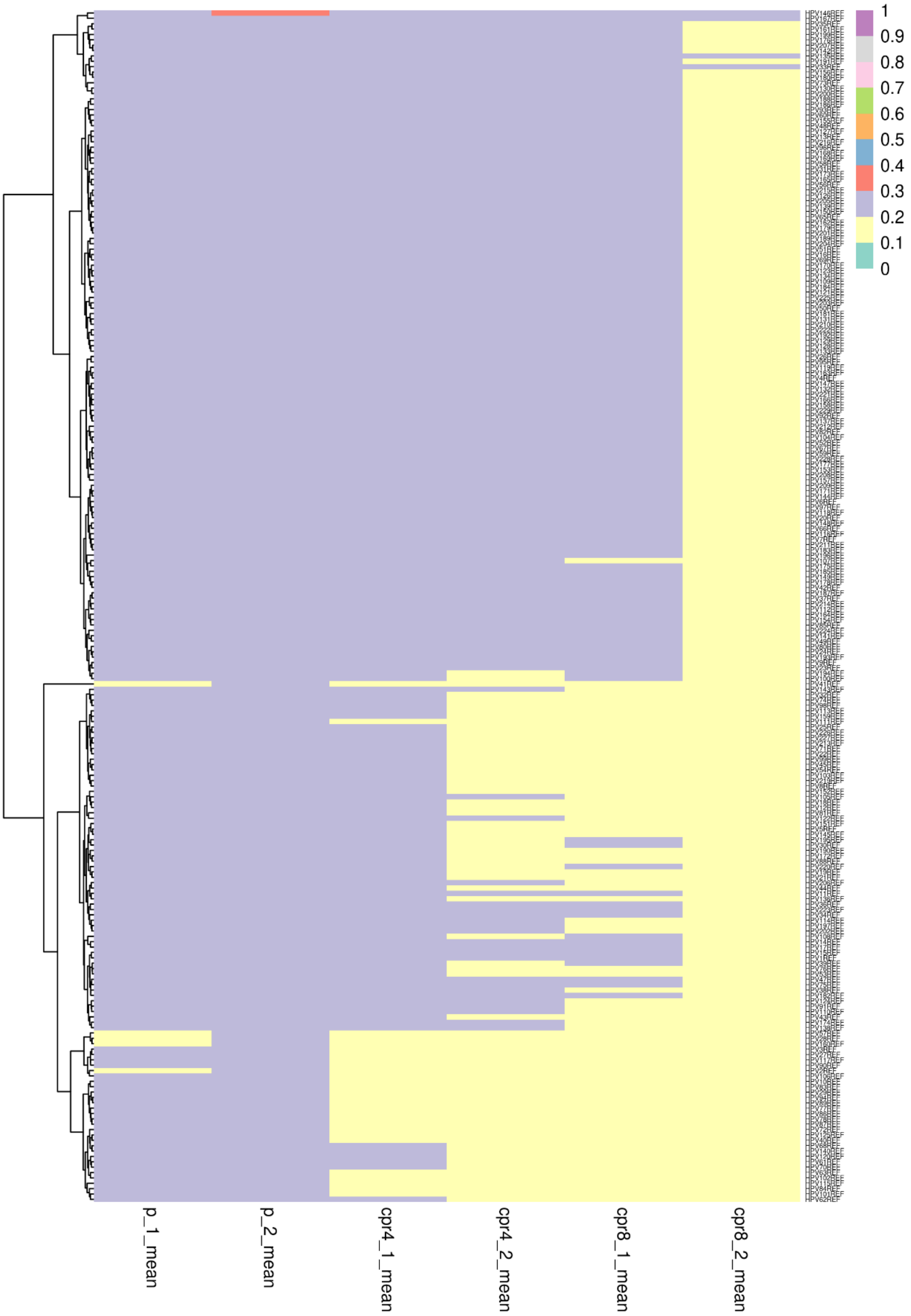

Figure S4. Heatmap of HPV-KITE similarity scores for HPV reference genomes in RNA-seq data from SCC-9 parental (P) and cisplatin-resistant (CPR4 and CPR8) cell lines replicates. Low (≤ 0.3) and high (> 0.3) scores are shown based on color distribuition.

Figure S5

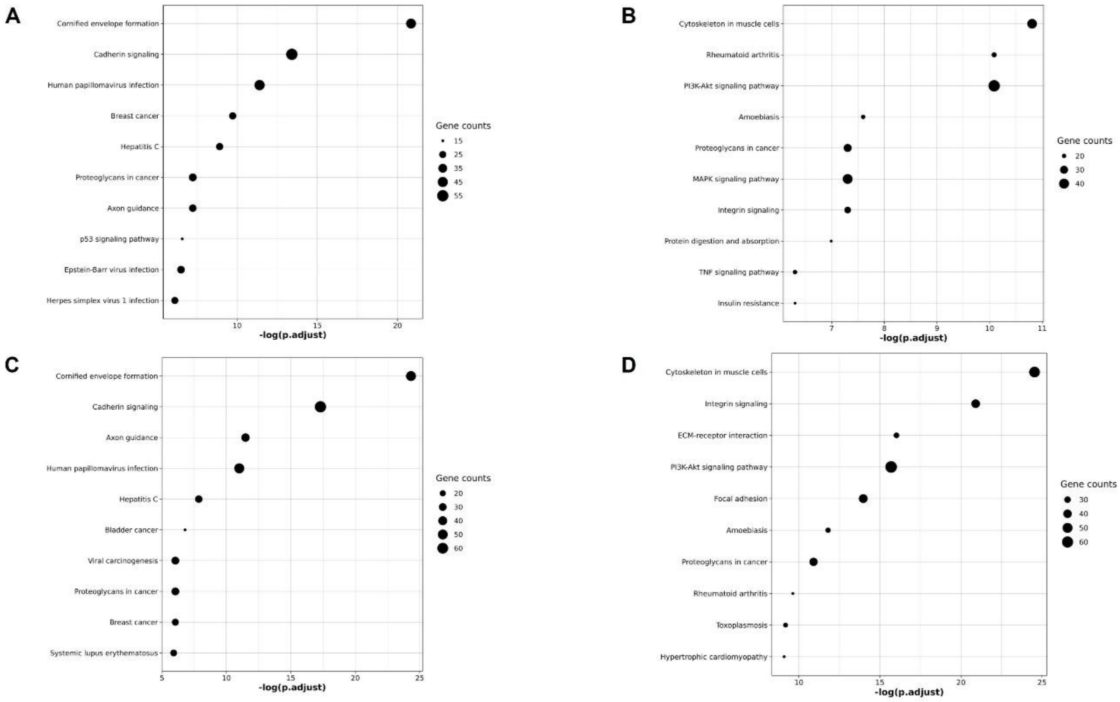

Figure S5. Kyoto Encyclopedia of Genes and Genomes (KEGG) pathways according to expression status in CPR4 and CPR8 resistant cell lines. The size of the bubbles in the plot represents the number of enriched differentially expressed genes (DEGs) in each pathway, the Y-axis shows 10 relevant pathways and X-axis label represents -log(p.adjust) value. (**A**) Bubble plot to enriched KEGG pathway downregulated CPR4 genes, (**B**) upregulated CPR4 genes, (**C**) downregulated CPR8 genes, and (**D**) upregulated CPR8 genes.

Figure S6

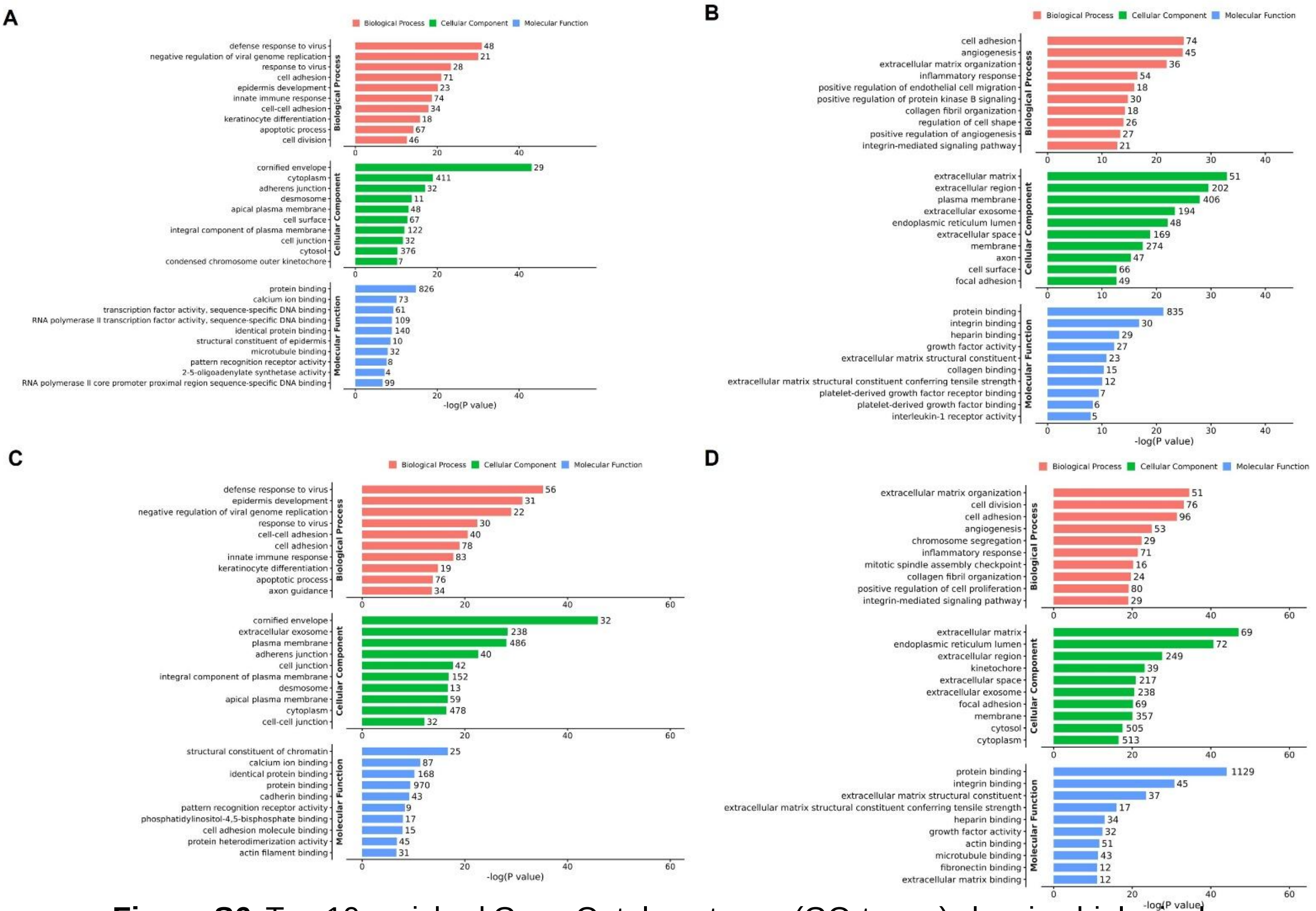

Figure S6. Top 10 enriched Gene Ontology-terms (GO-terms) showing biological process, cellular component, molecular function to expression status of resistant cell lines. (**A**) GO-terms enriched downregulated CPR4 genes, (**B**) upregulated CPR4 genes, (**C**) downregulated CPR8 genes, and (**D**) upregulated CPR8 genes.

Figure S7

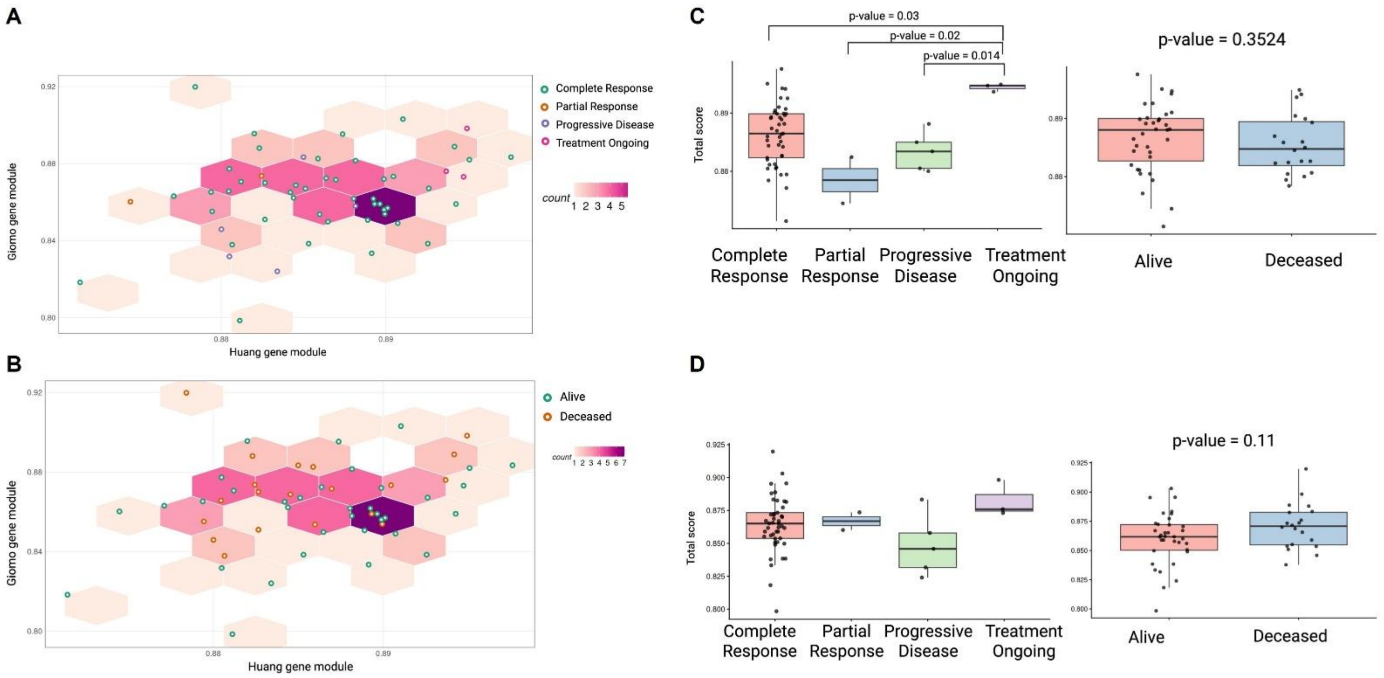

Figure S7. External evaluation of resistance-associated gene modules in cisplatin-treated TCGA-HNSC patients. (**A**,**B**) Scatter plots showing the relationship between the candidate 30-gene resistance-associated module score identified in the present study and the Huang platinum-resistance module score in TCGA-HNSC patients treated with cisplatin-based regimens. Each point represents one patient and is colored according to treatment outcome (**A**) or vital status (**B**). Group-specific convex hulls enclose the outermost observations within each clinical category. (**C**,**D**) Boxplots showing the distribution of the Huang platinum-resistance module scores (**C**) and the candidate 30-gene module scores (**D**) according to treatment-outcome category and vital status. Individual points represent patients, and exact *p*-values are shown.

Supplementary Materials

Table S1. Primer sequences used for RT-qPCR analysis

Gene name	Forward 5' – 3'	Reverse 5' – 3'	Product (bp)	Tm (˚C)
ZEB1	GCTTTTCCCATTCTGGCTCCTA	TCTTGGTCGCCCATTCACA	81	60
ZEB2	CACACACATACACAGAAAGGA	ATAACAGGAGGCATAGCATT	157	60
N-cadherin	GCGTCTGTAGAGGCTTCTGG	GCCACTTGCCACTTTTCCTG	293	60
E-cadherin	ACAGCCCCGCCTTATGATT	TCGGAACCGCTTCCTTCA	56	60
Vimentin	GACGCCATCAACACCGAGTT	CTTTGTCGTTCGTTAGCTGGT	248	60
SNAIL1	GCGTGTGCTCGGACCTTCT	ATCCTGAGCAGCCGGACTCT	67	60
SNAIL2	GGAGCATACAGCCCCATCA	TGGGTAGCTGGGCGTGGAA	61	60
TWIST1	AAGCTGAGCAAGATTCAGACC	CGTGAGCCACATAGCTGC	118	60
TWIST2	GCAAGAAGTCGAGCGAAGAT	GCTCTGCAGCTCCTCGAA	92	60
